# Roadway Runoff Induced Acute Mortality in Juvenile Coho Salmon During Spring Storm Events

**DOI:** 10.1021/acs.est.5c13992

**Published:** 2026-01-05

**Authors:** Marlee L. Brown, Nathan Ivy, Melissa Gonzalez, Justin B. Greer, John D. Hansen, Edward Kolodziej, Jenifer K. McIntyre

**Affiliations:** † Center for Urban Waters, Tacoma, Washington 98421, United States; ‡ Interdisciplinary Arts and Sciences, 43339 University of Washington Tacoma, Tacoma, Washington 98421, United States; § Department of Civil and Environmental Engineering, 7284 University of Washington, Seattle, Washington 98195, United States; ∥ Puyallup Research and Extension Center, Washington State University, Puyallup, Washington 98371, United States; ⊥ U.S. Geological Survey, Western Fisheries Research Center, Seattle, Washington 98115, United States

**Keywords:** Juvenile coho salmon, spring salmon, mortality, roads, 6PPDQ

## Abstract

Extensive mortalities of adult coho salmon (*Oncorhynchus kisutch*), often called “Urban Runoff Mortality Syndrome” (URMS), have been documented during the fall in creeks where water quality has been degraded by roadway runoff. The primary cause of mortality is 6PPD-quinone (6PPDQ; *N*-(1,3-dimethylbutyl)-*N*′-phenyl-*p*-phenylenediamine quinone)–an ozone transformation product that forms on all vehicle tires. Laboratory studies have shown that juvenile coho salmon are highly sensitive to 6PPDQ exposure. Unlike adults, juveniles reside in impacted watersheds year-round, including during the spring when 6PPDQ concentrations can frequently exceed lethal thresholds during storms. To assess the potential incidence of URMS in springtime rearing habitats for juvenile coho salmon, we conducted a paired water quality and toxicology study at Miller Creek, a runoff-impacted watershed in Normandy Park, WA, USA. Using a small field facility, three naïve groups of juvenile coho salmon (*N* = 720) were exposed to either creek water or groundwater (*N* = 120 per treatment per storm), across three spring storms while comparing water quality and mortality end points. In creek water during exposures, peak 6PPDQ concentrations reached 73–110 ng/L, exceeding reported median lethal concentrations (LC_50_) for coho salmon. Over each 24–73 h storm exposure period, ∼80% of Miller Creek-exposed juvenile salmon died. No mortality occurred among control fish exposed to groundwater. These results indicate previously unidentified mortality risks for juvenile life stages of coho salmon during spring storms, suggesting substantial and year-round water quality impediments to coho salmon health and recovery across roadway runoff-impacted spawning, rearing, and migratory habitats.

## Graphical abstract

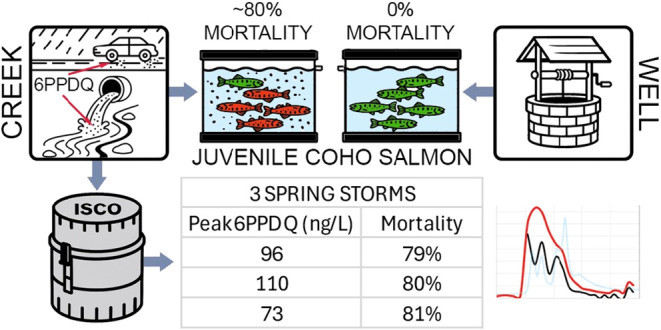

## 1 Introduction

With increasing urbanization, salmon-bearing watersheds across the Pacific Coast of North America are widely impacted by roadway runoff during storms, introducing a complex mixture of poorly defined contaminants into sensitive aquatic ecosystems.^1–4^ Adult coho salmon (*Oncorhynchus kisutch*) returning to lowland creeks subject to urbanization now have a well-documented history of stormwater-linked acute mortality events, sometimes known as “Urban Runoff Mortality Syndrome” (URMS)^5^ or “coho prespawn mortality”.^6^ Observations of symptomatic or dead adult coho salmon after storm events were first systematically documented in urban watersheds of the Pacific Northwest with “restored” (i.e., improved) physical habitat starting in the 1990s.^6,7^ In the most impacted watersheds, annual mortality rates for adult coho salmon can consistently exceed 90% from fall storm exposures.^6,8^ Such loss of adult spawners prior to successful reproduction could result in localized extinctions within the most impacted populations over the next century.^8,9^

URMS was first correlated to traffic^8^ and tire rubber leachate,^10^ and subsequently, the primary causal toxicant responsible for URMS was identified as 6PPD-quinone (6PPDQ; *N*-(1,3-dimethylbutyl)-*N*′-phenyl-*p*-phenylenediamine quinone).^11,12^ 6PPDQ forms when the rubber antioxidant 6PPD reacts with atmospheric ozone; this reaction protects the rubber from ozone damage.^13^ All vehicle tires globally contain 0.4–2.0% of 6PPD by mass (e.g., 40–100 g per passenger vehicle tire).^14,15^ As 6PPD diffuses up to air-rubber surfaces, 6PPDQ can be continuously generated,^16^ with yields of ∼1%, and released into the environment.^11,17^

The environmental impacts of the extensive roadway systems of human society are defined in part by the continual and abundant generation of tire wear particles from tires by roadway abrasion, as well as leaching of associated additive chemicals. As one example, 6PPDQ easily leaches from water-exposed rubber surfaces^18,19^ and therefore is widely detected in roadway environments and roadway-impacted systems, including water, dust, air, roadside soil, sediments and biota.^20,21^ In receiving waters, 6PPDQ concentrations are reported globally with concentrations up to ∼200 ng/L in Canada,^22^ 88 ng/L in Australia,^23^ and 140 ng/L in Norway.^24^ In roadway runoff, concentrations up to 2400 ng/L have been reported in Hong Kong and 2430 ng/L in China.^25^ In smaller receiving waters typical of coho salmon spawning and rearing habitats, 6PPDQ levels have been reported up to 450 ng/L in the Pacific Northwest,^26^ although peak event concentrations of 20–200 ng/L are more typical for small roadway-impacted creeks.^22,27,28^ Runoff-derived contaminant concentrations in receiving waters are dynamic, with peak concentrations typically aligning with maximum discharge and lasting only a few hours.^29^

Juvenile coho salmon fry emerge from streambed gravel nests (“redds”) throughout late winter and early spring, coinciding with seasonal storms that potentially introduce 6PPDQ into rearing habitats. Coho salmon juveniles then spend roughly one year inhabiting freshwater systems, often including low flow headwater and small tributary habitats, before migrating through estuaries to the ocean. Median 24 h lethal concentrations (LC_50_) for juvenile coho salmon range from 41–95 ng/L;^27,29–31^ such concentrations are frequently exceeded in smaller streams receiving roadway runoff that lack substantial dilution capacity.^22,26,28^ As a result of coho salmon sensitivity to 6PPDQ, the U.S. Environmental Protection Agency released an Acute Aquatic Life Screening value of 11 ng/L for 6PPDQ.^30^ Importantly, we have detected concentrations of 6PPDQ in coho salmon rearing habitats during spring storm events similar to those observed in fall storms affecting adult coho salmon spawners, implying that poor water quality—evidenced by high 6PPDQ concentrations—is a year-round phenomenon in roadway impacted systems.^10^

To the best of our knowledge, systematic observations of URMS events in juveniles have not been documented, possibly because juvenile salmon are generally difficult to observe due to their small size and cryptic coloration and behavior.^31^ However, anecdotal observations of storm-associated mortality in juvenile salmonids in spring storms have been reported in British Columbia (Paul Cipywnyk, Byrne Creek Streamkeeper, personal communication Apr. 28, 2022; ZoAnn Morten, Pacific Streamkeepers Federation, personal communication May 8, 2025) and on Miller Creek (Iris Kemp, King County, personal communication Apr 9, 2024). Together, these anecdotal mortality observations suggest that URMS may pose substantial undocumented risks to juvenile coho salmon present in rearing and migratory habitats.

Coho salmon are an economically, ecologically and culturally important species of fish that are increasingly threatened by roadway runoff impacts to spawning and rearing habitats. This study investigated URMS and 6PPDQ risks to juvenile coho salmon health in a representative rearing habitat during spring storm events. To assess URMS risks to juvenile coho salmon, we conducted a paired water quality-ecotoxicology study at a small runoff-impacted creek in Washington State (Miller Creek, Burien/Normandy Park, Washington State, USA) where substantial URMS has been documented for returning adult salmon during the fall,^10^ and where we have previously documented water quality impairment and high 6PPDQ concentrations during storms.^10,11,29,31^ Creek eDNA surveillance indicated the presence of coho salmon and coastal cutthroat trout (*Oncorhynchus clarkii clarkii*) through all reaches; important given that coastal cutthroat trout is the second most sensitive species for 6PPDQ.^32^ Over three spring storms (April-June 2024), juvenile coho salmon were exposed to either runoff-impacted creek water or groundwater as a control. Concentrations of 6PPDQ and other roadway chemicals were measured using liquid chromatography-tandem mass spectrometry and coho survival was monitored across the exposure periods.

## 2 Materials and Methods

### 2.1 Chemicals

PPD transformation products and other analyte stocks were prepared as described previously.^33^ PPD stocks were prepared in methanol (LCMS grade, Fisher Scientific, Waltham MA, USA) from analytical standards using deionized water (Milli-Q Ultrapure) and stored in airtight bottles in bags at 4 °C. The prepared stocks along with the calibration standards were stored at −20 °C and replaced every 2 months to limit instability effects. A complete list of chemicals is provided in the Supporting Information (Table S1).

### 2.2 Site and Sampling

Miller Creek is a small residential, commercial and urbanizing watershed (∼2070 ha) with multiple inputs of treated and untreated roadway runoff that induce high rates of storm-linked mortality in adult coho salmon annually.^10,11,29,31^ Paired exposure studies were conducted in a small field laboratory facility (Figure S1) adjacent to Miller Creek, (coordinates 47.442236, −122.326659). There is no wastewater discharge in Miller Creek; therefore, water quality during storms is driven by roadway runoff and other stormwater inputs. The modified Mediterranean climate of western Washington typically has nearly dry, warm summers followed by frequent low-moderate intensity precipitation throughout the October-May period. Regionally, spring and late spring storms tend to be lower intensity and smaller, and often follow longer dry periods, relative to characteristics of fall season storms.

Water samples were collected from Miller Creek ∼20 m from the field laboratory and ∼1 m upstream of the pump supplying creek water to the field laboratory using an automated water sampler (Teledyne ISCO 6712, Lincoln, NE USA; see Figure S1). Twenty-four 350 mL glass bottles were used to collect and composited into 12 2–4 h time-dependent 640 mL samples throughout sampling events. Baseflow samples consisted of 20–40 mL aliquots every 15–30 min over 2 h in bottles 1 and 2. Storm sampling compositing generally consisted of 40–80 mL aliquots sampled every 15–30 min over 1–1.5 h in bottles 3–24. Sampling began in baseflow periods 4–6 h prior to storms and continued through the storm hydrograph until all bottles were filled. Three baseflow events (dry periods between storms without measurable rainfall) were sampled with an antecedent dry period (ADP) greater than 2 days on April 16 (6 days ADP), May 14 (6 days ADP), and June 26, 2024 (8 days ADP). Three storms used for fish exposures initiated by forecasted precipitation were sampled on April 25 (3 days ADP), May 21 (2 days ADP), and June 2, 2024 (2 days ADP). The first two storms generated 12 composite water samples over 26 h, the third storm was longer and generated 24 composite samples over 73 h (Figure S2). For groundwater controls, collected from the influent plumbing into raceway A, 1 L grab samples were collected before and after storms (*N* = 4) from the exposure tank feed. Similar 1 L grab samples were collected from Miller Creek before and after each storm and baseflow sampling event (*N* = 13) to define pre- and poststorm concentrations.

### 2.3 Hydrological Data

Hydrology data for flow and to calculate cumulative discharge and contaminant mass loads were collected from an automated gage (stream gauge 42A; 47.44548, −122.35196; King County Hydrologic Monitoring Program) located within 0.6 km of the field laboratory facility and study site. Precipitation data were collected from the nearest automated station reported by Weather Underground (Rabbit Hill—KWASEATT2555, Burien, WA 47.46700, −122.33400) and located 2.7 km away. Targeted storms were selected based on a rainfall forecast of >6 mm and ADP > 2 days.

### 2.4 Fish Exposures

Juvenile coho salmon were acquired from Soos Creek Hatchery (Washington State Department of Fish & Wildlife) prior to each of the three targeted storm events. Fish were age 0+ (mass: 2.6 ± 0.8 g) (mean ± standard error of the mean; SEM), length 62.4 ± 5.7 mm (mean ± SEM). Thirty fish were placed in each of 8 glass aquaria containing 32 L of well water. Each aquarium was perforated with a mesh-covered outlet (12.7 mm diameter) enabling water to flow-through at 70 ± 5 L/h, therefore maintaining fish loading below the recommended maximum of 0.5 g/L/day (OECD Test Guideline 203). The 8 aquaria were divided among two fiberglass raceways (Figure S1) that were supplied with flow-through creek water to standardize temperatures across treatments. Upon arrival, fish were acclimated for a minimum of 24 h and subsequently fed commercial pellets (2.5% body weight) every other day. Daily care included removal of uneaten food and feces, monitoring for clinical signs and survival, and measuring basic water quality, including water temperature, dissolved oxygen, pH and conductivity.

Within 3 h of storm initiation, inflow waters to the aquaria of one raceway were switched from well water to flow-through water pumped directly from Miller Creek. Fish were not fed the day of a storm pulse and food was withheld until completion of the storm and turbidity subsided (3 days postexposure for Storms 1 and 2, 3.5 days for Storm 3). Fish were monitored for survival, signs of stress (flashing, erratic swimming, color change, lethargy) and symptoms of URMS (surface gaping, surface swimming, loss of equilibrium) during exposures. Dead fish were promptly removed and recorded. Fish showing clinical signs of URMS^34^ were removed and euthanized by a lethal dose of MS–222 (500 mg/L buffered to pH 7). Lengths and weights were recorded for mortalities and euthanized survivors at the end of each storm exposure. Animal care and euthanasia methods were approved by the Washington State University Institutional Animal Care and Use Committee (ASAF#7134).

### 2.5 Statistical Analyses

Differences in survival among the three storm events were assessed using Kaplan–Meier survival analysis in R (version 4.4.3), with survival monitored at 24 h intervals and treated as interval-censored. Survival objects were constructed with the Surv­() function, and survival curves were estimated using survfit­() from the *survival* package. Group differences were tested using the survdiff­() function (log-rank test). When a significant overall effect was detected, post hoc pairwise comparisons were conducted using the pairwise_survdiff­() function from the *survminer* package, with a Bonferroni correction applied for multiple comparisons. Kaplan–Meier survival curves were visualized using ggsurvplot­() from the *survminer* package. Daily water quality was assessed in R with a linear mixed effects model using the lmer­() function from the *lmerTest* package with storm number as a random variable.

### 2.6 Contaminant Analysis

Water samples were transported to the Center for Urban Waters (Tacoma, WA) on ice and processed within 24 h. Samples were combined for composites and split into duplicates. Samples (200 mL) were processed under conditions previously reported.^27^

6PPDQ and select other stormwater contaminants, including various PPDs and other 6PPD transformation products, were quantified on an Agilent 1290 Infinity II HPLC system coupled with a 6495D triple quadrupole MS/MS instrument (LC-MS/MS) using an expanded version of the liquid chromatography-triple quadrupole mass spectrometry method reported by Hou et al.^33^ Dynamic multiple reaction monitoring (dMRM) with 2–3 transition ions was used to quantify and confirm analytes. Calibration range, internal standards, and method detection/quantification limits are provided in Supporting Information (Table S1).

### 2.7 Quality Assurance and Quality Control

QA/QC for baseflow and targeted storms consisted of method blanks, field blanks, deionized water spikes, and matrix spikes (Table S2). Field blanks (*N* = 6) consisted of 1 L of Milli-Q water pumped through the ISCO and processed identically; method blanks (*N* = 6) consisted of 200 mL Milli-Q water extractions. Due to a sample contamination issue from cleaning, 6PPDQ was detected in one method blank at 3.7 ng/L and one field blank at a concentration less than the method quantification limit. PPDs, transformation products and other analyte detections are reported in Table S3. Detections in control samples in groundwater source are reported in Table S4. Background samples collected from the creek water before and after a storm are reported in Table S5. Matrix spikes (*N* = 12) consisted of spiking analytes into creek water at either 125 or 375 ng/L. 6PPDQ recovery in matrix spikes was 99 ± 4.1%, 6PPDQ recovery in deionized water spikes (*N* = 7, 125 ng/L) was 97 ± 2.7%. Spike-recovery data in laboratory controls and matrix spikes are reported in Table S6, respectively. Blanks and spike recovery samples were analyzed in duplicate while deionized water spikes were in triplicate; results of replicate analysis for 6PPDQ indicated <10% coefficient of variation.

### 2.8 Runoff and Mass Loads

To create hydrographs and pollutographs, normalized cumulative precipitation values were calculated (accumulation, in mm, eq 1). Normalized cumulative runoff volumes were calculated from archived discharge values (*Q*, in m^3^/s; eq 2); calculations began at the time point when observed discharge values increased. Normalized contaminant mass loads (eq 3) were calculated using measured contaminant concentrations (*C*_*t*_; in ng/L) over the sampled period. Mass load calculations and curves match those reported by Peter et al..^29^

1 $$normalized\;cumulative\;precipitation\;volume\;\left(in\,\;\%\right)=\frac{\left({accumulation}_{t}-{accumulation}_{t_{0}}\right)}{\left({accumulation}_{t,final}-{accumulation}_{t_{0}}\right)}\times 100$$

2 $$\mathrm{normalized}\,\;\mathrm{cumulative}\;\mathrm{run}\;\mathrm{off}\;\mathrm{volume}\;\left(\mathrm{in}\;\%\right)=\frac{\sum_{t_{0}}^{t}\left(Q_{t}-Q_{\min}\right)\Delta t}{\sum_{t_{0}}^{t_{\mathrm{final}}}\left(Q_{t}-Q_{\min}\right)\left(t_{\mathrm{final}}-t_{0}\right)}\times 100$$

3 $$\mathrm{normalized}\;\mathrm{cumulative}\;\mathrm{conta}\,\mathrm{minant}\;\mathrm{mass}\;\mathrm{load}\;\left(\mathrm{in}\;\%\right)=\frac{\sum_{t_{0}}^{t}C_{t}Q_{t}\Delta t}{\sum_{t_{0}}^{t_{\mathrm{final}}}\left(C_{t}\right)\left(Q_{t}-Q_{\min}\right)\left(t_{\mathrm{final}}-t_{0}\right)}\times 100$$

### 2.9 eDNA Analysis

Surveys of eDNA were performed quarterly (July 27, 2023; November 29, 2023; February 23, 2024; and May 31, 2024) at four longitudinally spaced sites on Miller Creek. eDNA sampling followed the protocol of Ostberg et al. (details in Supporting Information). These data surveys extend the population risk for spring storms.

## 3 Results and Discussion

### 3.1 Hydrology

Between April 1–June 30, 2024, 16 precipitation events occurred in Miller Creek where flows at least doubled from baseflow conditions; most storms were low intensity and lasted only 1–3 h (Figure S2). Baseflow discharge in Miller Creek ranged from 0.05–0.1 m^3^/s. Baseflows in this rainfall dependent watershed are generally linked to the length of antecedent dry periods, with shorter antecedent dry periods generating higher baseflows. Targeted storm events required a predicted rainfall >5 mm and an antecedent dry period >48 h for fish exposures. Antecedent dry periods prior to the three targeted storms were 3, 2, and 2 days for Storms 1–3, respectively. The antecedent storm occurring on April 21, 2024 (preceding targeted Storm 1 on April 25) had notably high rainfall of 19.6 mm. The other two antecedent storms (May 18/19 and May 29, 2024) preceding targeted Storms 2 and 3 on May 2 and June 2–3, respectively, were small (0.3–8.1 mm), low intensity events lasting only 1–2 h and with only <0.2 m^3^/s changes to creek discharge.

Targeted Storm 1 (20.8 mm precipitation, 12.7 mm/h maximum intensity), Storm 2 (17.3 mm precipitation, 9.4 mm/h maximum intensity), and Storm 3 (22.9 mm precipitation, 11.9 mm/h maximum intensity) increased flows to 0.93, 1.1, and 1.4 m^3^/s, respectively. These flows were about 10-fold higher than their preceding baseflows; these were the three largest storms during this period (Figure S2). Storms 1 and 2 reflected relatively common spring storm events in size and duration. The June 2 storm (i.e., Storm 3), with two major pulses of precipitation and 2 days of substantially higher flows, represented a somewhat unusually large and long storm for a late spring-early summer event.

### 3.2 Water Quality

Daily water quality was similar between the well water and the streamwater across the three fish exposures (Table S7). Only pH was significantly different (*t*(105) = −2.509, *p* = 0.013) between the well and streamwater, with a slightly reduced pH in well water (AVG ± standard deviation (SD): 7.83 ± 0.26) compared with the streamwater (7.94 ± 0.19).

In composite samples of baseflow conditions between and after the targeted storms, 6PPDQ concentrations in Miller Creek averaged 1.1 ng/L, 1.0 ng/L and 1.8 ng/L, respectively (Tables S8–S10). In 10 of 13 complementary grab samples collected before and after baseflow and storm events (processed in duplicate) 6PPDQ was <4.0 ng/L; two after-storm grabs and one before-baseflow grab had concentrations >15 ng/L due to smaller rain events before and after the targeted baseline event (Table S5). 6PPDQ concentrations detected in groundwater control samples of 6PPDQ were <0.3 ng/L.

During the three targeted storms, 2–3 h composite samples were collected over 26–73 h producing 12–24 samples for each storm. 6PPDQ was detected in 98% of Miller Creek storm samples (*N* = 48). 6PPDQ concentrations rapidly rose as streamflow rose, with peak concentrations reaching 96, 110, and 73 ng/L (average: 93 ng/L) for the three targeted storms, respectively (Figure 1A–C). Peak composite 6PPDQ concentrations tended to occur within 2–4 h after stream discharge increased, but for the second targeted storm event with two separate waves of precipitation, the peak 6PPDQ concentration occurred 8–9 h later and was concurrent with the second peak in the hydrograph. Because concentrations of roadway-derived contaminants in small watersheds can vary over time scales of minutes, these composites likely underestimated peak 6PPDQ concentrations in the creek but are likely reflective of biological exposure dynamics.^22,29,35^

**Figure 1:**
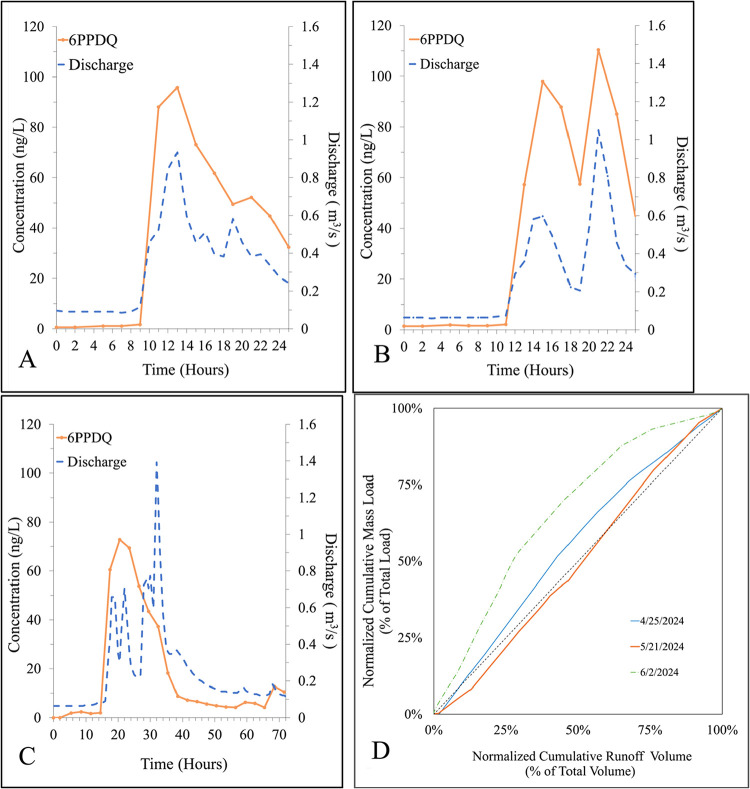
Observed 6PPDQ concentrations and hydrographs over 24 h for Miller Creek during targeted storm events: (A) April 25, 2024 (Storm 1), (B) May 2, 2024 (Storm 2), and (C) June 2–3, 2024 (Storm 3). Panel (D) plots normalized cumulative 6PPDQ mass load (as % of total load) against cumulative runoff volume (as % of total volume). The 1:1 dashed line represents uniform rates of mass transport and runoff volume.

After 6PPDQ concentrations and hydrographs peaked, 6PPDQ concentrations slowly declined to 10–40 ng/L, although not dropping to the <5 ng/L levels typical of baseflow within the sampled time frame. 6PPDQ concentrations exceeded the 11 ng/L and 12 ng/L EPA and WA ECOLOGY Aquatic Life Screening criteria^30,36^ for greater than 16, 14, and 27 h for Storms 1–3, respectively. Measured values also exceeded the juvenile coho salmon LC_50_ value of 41 ng/L LC_50_ reported by Lo et al.^37^ for 14, 14, and 15 h respectively, and exceeded the 80 ng/L LC_50_ value of Greer et al.^38^ for 4, 8, and 0 h, respectively (Table S11). Concentrations of other roadway-derived contaminants were also analyzed in these samples; including PPDs (Table S12), 6PPDQ and other transformation products (Table S13), and other vehicle related chemicals (Table S14).

We recently reported a multiseason, multiyear contaminant data set for storm events (*N* = 17) in Miller Creek,^31^ and other studies also have reported similar 6PPDQ concentrations and contaminant dynamics,^22,23,39^ especially for smaller urbanizing watersheds similar to Miller Creek. In hourly composites, 6PPDQ was present up to concentrations of 79–164 ng/L, with similar temporal dynamics, across three small runoff-impacted creeks in British Columbia, Canada.^22^ 6PPDQ reached 88 ng/L in a tributary impacted by roadway runoff (160–1800 m downstream of two major roadways) of the Brisbane River in Australia.^23^ Samples collected across the United States detected 6PPDQ in 57% of stormwater samples and 45% of “urban-impacted” receiving water samples at concentrations of 2–290 ng/L.^39^ In the Great Lakes region, 6PPDQ was present in 80% of roadway-impacted tributary samples at concentrations up to 82 ng/L.^28^ Notably, many such reported concentrations in receiving waters were near or above LC_50_ values for sensitive species where some mortality would be expected in exposed populations.^32,37,38^

Contaminant mass and transport dynamics depend heavily on weather and watershed characteristics such as size, shape, chemical properties, temperature, land use, hydraulic alteration, and receiving water volumes available to support dilution.^22^ Despite the ∼50% variation in peak concentration (73 to 110 ng/L), the estimated mass loads (1900–2100 mg) of 6PPDQ (Figure 1D) were similar (within ∼10%) for each of the three storms. In a preceding study conducted across 17 storms in Miller Creek during 2020–2023, 6PPDQ mass loads ranged from 78–2700 mg/storm, with an average load of 980 mg and median load of 760 ± 770 mg.^31^ By load, the three targeted spring storms of this study were among the larger mass transport events yet observed for Miller Creek. Mass load estimates for all contaminants are reported in Table 1.

**Table 1:** Observed Detection Frequencies (*N* = 48 Storm Samples), Concentration Ranges, Peak Concentrations and Estimated Mass Loads for PPDs, PPDQs, 6PPD Transformation Products (TPs) and Vehicle Related Contaminants in Miller Creek across the April 25, May 20, and June 2, 2024 Storms^a^

contaminant/class		detection frequency (%)	concentration range (ng/L)	peak concentration (ng/L)	mass loads per storm (mg)
PPDs	6PPD	75	<MDL–360	52–360	1300–5300
	IPPD	19	<MDL–4.9	3.6–4.9	<MDL–90
	7PPD	2	<MDL–0.6	<MDL–0.6	<MDL–40
	DPPD	40	<MDL–120	8.9–120	200–1200
	DTPD	42	<MDL–83	17–83	500–1400
	DNP	0	<MDL	<MDL	<MDL
∑PPDS			**<MDL–570**		**2000–8000**
PPDQs/TPs	6PPDQ	98	<MDL–110	73–110	1900–2100
	IPPDQ	0	<MDL	<MDL	<MDL
	7PPDQ	0	<MDL	<MDL	<MDL
	DPPDQ	0	<MDL	<MDL	<MDL
	DTPDQ	15	<MDL–52	<MDL–52	<MDL–1200
	1,3-DMBA	90	<MDL–2700	340–2700	11,000–44,000
	4-OH-DPA	81	<MDL–140	85–140	1700–2700
	4-ADPA	40	<MDL–470	<MDL–470	<MDL–7600
	4-DPA	71	<MDL–510	170–510	3500–9100
	4-NDPA	71	<MDL–20	7.0–20	1500–2700
∑PPDQs/TPs			**<MDL–4000**		**20,000–67,000**
vehicle related contaminants (VRCs) ∑VRCs	DPG	100	50–1300	950–1300	23,000–29,000
	HMMM	100	24–1200	750–1200	18,000–25,000
	NCBA	71	<MDL–18	14–18	300–400
	1-H-BTR	100	10–550	280–550	5200–9900
	5-Me-1-H-BTR	100	30–1700	970–1700	19,000–28,000
	2-NH2-BTH	98	0.7–91	87–91	1600–2500
	2-OH-BTH	100	34–240	210–240	4600–7100
	2-Mo-BTH	94	1.5–40	15–40	300–500
			**150–5100**		**72,000–102**,**000**

a Concentrations for PPDs are considered semi-quantified due to their instability, <MDL represent concentrations below the method detection limit (Table S1).

Predictable relationships between storm conditions, contaminant sources, transport mechanisms, and concentrations or mass loads remain challenging.^31^ In our data set, 6PPDQ dynamics across three storm events were most consistent with continuing and increasing mass transport throughout the storms (Figure 1D). During Storm 1 and Storm 2, cumulative 6PPDQ mass loads either somewhat exceeded or nearly mirrored cumulative runoff volumes, with middle or mixed flush dynamics over the storms. The larger and longer June 2 storm was more typical of a middle flush dynamic.^40^ In these storms in this watershed, continued precipitation resulted in continued and even growing 6PPDQ mass transport relative to streamflow as hydraulic connectivity and mass transport pathways were established that mobilize contaminant mass.^41^ Notably, no clear evidence of dilution with continued precipitation or streamflow was apparent in these data, indicating that even in late spring, after dozens of fall-winter-spring storm events had occurred, substantial 6PPDQ mass remained available for environmental transport. While some roadway-impacted watersheds do not exhibit such transport-limited behavior, it is likely that the more ‘urban’ or ‘urbanizing’ watersheds, or the more highly trafficked locations, have accumulated substantial reservoirs of roadway and vehicle-derived contaminants that are difficult to deplete during multiple precipitation-induced transport events.^22^

Concentration trends and dynamics for other roadway and vehicle derived contaminants analyzed concurrently with 6PPDQ also were evaluated. Pollutographs and pollutographs as normalized cumulative mass loads and runoff volume for PPDs (Figures S3–S6), 6PPD transformation products (Figures S7–S10), and for other vehicle related compounds (Figures S11–S15) were developed and were broadly similar to those reported here for 6PPDQ and with prior studies reporting Miller Creek contaminants.^10,29,31^ Pollutographs show many roadway and tire-derived contaminants, including various other PPDs, PPD-quinones, and transformation products of 6PPD, are copresent with 6PPDQ and often reflect similar chemodynamics, with increasing concentrations and mass transport with discharge and time. Pollutographs as normalized cumulative mass loads and runoff volumes are relative to the proportional rate of contaminant mass transport to runoff volume across the storm in the receiving water. In particular, the similar middle flush and transport-limited dynamics across these contaminants emphasize the common source, abundant mass, and similar chemical characteristics and structures of these contaminants.

As one example, hexamethoxymethylmelamine (HMMM), a chemical used in tire rubber and automotive plastics, also occurs in roadway runoff impacted waters and tends to exhibit hydrological profiles similar to 6PPDQ.^42^ In Miller Creek, HMMM concentrations ranged from 24–1200 ng/L, similar to previous reports in surface waters.^42,43^ Diphenylguanidine (DPG) is another widely detected and abundant (up to 1000s of ng/L in roadway-impacted surface waters)^15,44,45^ compound used in tire manufacturing. Miller Creek concentrations ranged from 50–1300 ng/L, similar to previous reports.^10,33^ Though we consider PPD parent concentrations semiquantitative, 6PPD concentrations (52–360 ng/L across the storms) largely mirrored those for 6PPDQ and spiked 2–4 h after the rain started (Tables S12–S13). PPD values may be underestimated due to instability and use of 6PPDQ-d5 as an internal standard for quantification.

Mass loads for analyzed contaminants ranged as low as 4 mg/storm and as high as 5300 mg/storm for PPDs, with a detection frequency of 0–75% across all storm samples. PPD transformation products ranged 1200–44,000 mg/storm with detection frequencies of 0–98%; mass loads for other vehicle related chemicals ranged 400–29,000 mg/storm with detection frequencies of 71–100% across the three storms (Table 1). Again, noting that storm sampling stopped before contaminant concentrations had returned to baseflow values, total PPD mass loads were at least 8000 mg/event, PPD transformation products were at least 67,000 mg/storm, and other vehicle-derived chemicals were at least 100,000 mg/storm (Table 1). Of these, 6PPDQ represented only 3.2% of the total detected PPD transformation product load mobilized across each event, with mass loads of 1,3 DMBA typically dominating detected contaminants. Per storm peak concentrations and mass loads for all chemicals are reported in Table S15.

### 3.3 Occupancy and Distribution of Fish Species

Prior to heavy urbanization, Miller Creek provided productive spawning and rearing habitat, with anecdotal reports of thousands of spawning coho salmon and chum salmon (*O. keta*) observed annually (Andy Batcho, Trout Unlimited, personal communication Sep 24, 2025). Since 2015, daily stream surveys report the yearly average return as 69 adult coho salmon (Iris Kemp, King County, personal communication Sep 23, 2025) which likely includes many adults for which Miller Creek was not their natal habitat. To better understand the occupancy, distribution and risk for fish species in Miller Creek, we conducted an eDNA survey for multiple reaches (Figure S16). As expected, sculpin spp. (*Cottidae*) were only found in the lower reach nearest Puget Sound likely due to food availability and barrier effects. Coho salmon were more frequently detected in the lower and mid reaches but found throughout, while coastal cutthroat trout were detected across all four reaches with highest detection rates associated with late winter/spring aligning with their spawning period.^46^ Consistent with their life history in fresh water, chum salmon were only detected within the lower reach in November 2024 (coincident with spawning) and subsequently in February 2025 (as emergent fry).

Importantly, electrofishing surveys (4% index reach) conducted in 2020 by King County Water and Land Resources Division indicated that the juvenile coho salmon population in Miller Creek was low (0.03–0.22 coho/m) compared with similar-sized, more rural streams (0.38–0.63 coho/m) and that survival to later summer was poor, with an estimated 3% survival from February to August (Chris Gregersen, King County Technical Memorandum, Dec 15, 2020). The estimated 20% survival through summer for coho salmon fry in more pristine watersheds^47^ supports that water quality could be a limiting factor in Miller Creek. This has serious implications for salmon conservation in systems like Miller Creek; for example, from 1981 to 2024, between 24,000 and 240,000 hatchery-spawned coho salmon fry were annually released to Miller Creek in early winter to aid in salmon conservation (Iris Kemp, King County, personal communication Sep 22, 2025). Similar to coho salmon, coastal cutthroat trout are also sensitive to 6PPDQ,^32^ and are found year-round throughout the creek indicating risk for this population as well. Thus, the impacts of 6PPDQ exposure to sensitive species may differ depending upon both the location and timing of juvenile residency and adult spawning events.

### 3.4 Coho Salmon Survival

No mortality was observed in any control replicates exposed to groundwater during the storm events. In contrast, each of the three storm exposures elicited significant mortality in juvenile coho salmon, with cumulative mortality rates of 79, 80, and 81% for Storms 1–3, respectively (Figure 2A–C). Kaplan–Meier analysis revealed significant differences in survival among the three storm events (*p* < 0.001). Pairwise comparisons indicated that Storm 2 showed a significantly different pattern (*p* < 0.001) of more protracted mortality across time (Figure 2B). Storms 1 and 3 were characterized by short, high intensity rain events. During these storms most mortality occurred over the subsequent 24 h. Storm 1 delivered 18 mm in 1 day, resulting in 79% mortality over the first 24 h; Storm 3 delivered 24 mm, resulting in 78% mortality within 24 h (Figure 2A,C). For these storms, we observed the onset of clinical signs (e.g., surface swimming, gaping) 13–15 h after precipitation began, 7–9 h after creek discharge increased (>15%), and 3–5 h after the peak 6PPDQ concentration (96 ng/L for Storm 1; 78 ng/L for Storm 3) was observed in composite samples. During Storm 3, symptomatic fish were observed during the rain event; however, due to high turbidity, the counting of deceased individuals did not occur until the following day.

**Figure 2:**
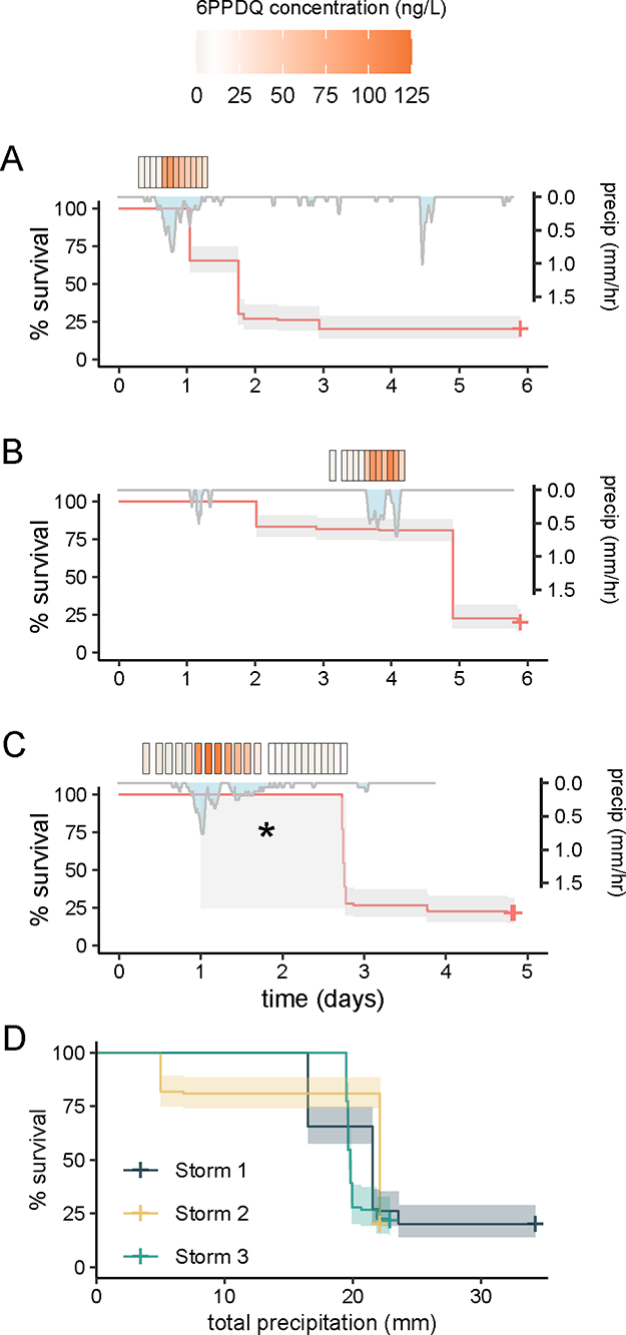
Observed mortality across time since fish exposure for juvenile coho salmon (*Oncorhynchus kisutch*) for Storms 1–3 (A–C) with corresponding precipitation and measured 6PPDQ concentration data. In (C), the gray box and * indicate that symptomatic fish were observed during this time period, but quantification of mortality was not possible due to high turbidity; all deceased individuals were counted on the following day. (D) Observed mortality from each storm as a function of total rainfall, shading reflects 95% confidence intervals. No mortality was observed in any groundwater controls.

The Storm 2 exposure period contained two distinct precipitation events. An initial small rain event (5 mm) induced 18% mortality, followed 3 days later by a second, larger 17 mm event that induced an additional 62% mortality. Across all 3 storms cumulative mortality was closely tied to cumulative precipitation (Figure 2D) that reflected the timing of increased runoff and the mobilization of 6PPDQ into Miller Creek (Figure 1). During Storms 1 and 3 mortalities increased with estimated mass of 6PPDQ mobilized into the stream up to approximately 1800 mg. Total 6PPDQ loads could not be calculated for Storm 2 due to missing concentration data from the initial rain event.

### 3.5 Environmental Implications

Previous studies have documented the impacts of fall storms on adult spawning populations of coho salmon; more recent studies have demonstrated that fry life stages of coho salmon and coastal cutthroat trout are extremely vulnerable to 6PPDQ, with 24 h static LC_50_ estimates of 38–41 ng/L for fry.^32,37^ Here, we observed composite, maximum concentrations for 6PPDQ during three spring storms in a salmon-bearing watershed that were at or above-reported LC_50_ values for similarly staged juvenile coho salmon (LC_50_: 80 ng/L [CI: 63–98])^38^ and far exceeding coho salmon fry LC_50_ (41 ng/L [CI: 34–48])^37^ values, with concurrent observations of 79–81% juvenile coho salmon mortality per storm exposure. These data indicate the potential for widespread and extensive mortality events in roadway runoff-impacted rearing and migration habitats for juvenile coho salmon that have gone undocumented. Juvenile coho salmon spend more than a year in freshwater habitats before outmigration, maximizing the probability that they will encounter one or more storms in watershed impacted by roadway runoff, resulting in mortality linked to 6PPDQ.This is in addition to the already low (16%) rate of salmon successfully transitioning from alevin to smolt stages in nonfragmented habitats.^47^

Given the potential for recurrent mortality events with high rates of lethality, it is likely that roadway-derived 6PPDQ exposures are changing coho salmon abundance and population structures in impacted roadway systems across the west coast of North America. For context, from 2019–2023, combined State, Federal and Tribal hatcheries released an average of 26 M coho salmon annually in Washington State alone. Additionally, 10 s–100 s of thousands of coho salmon fry are released annually into small creeks like Miller Creek by community or school groups focused on conservation and habitat efforts for wild salmon. The potential loss of significant numbers of juvenile hatchery fish after release due to impaired water quality is both expensive and inefficient. At the other end of their life cycle, approximately 700 K adult wild and hatchery reared coho salmon migrate from the ocean back to spawning grounds annually in the Puget Sound, almost all of which must transit roadways or roadway-impacted locations at some point during their migrations.

Based on these emerging water quality data around 6PPDQ occurrence, coho salmon mortalities are not a simple first flush or early fall storm phenomenon constrained to adult salmon, but rather a phenomenon better characterized as a year-round hazard for all life stages of fresh water residency for coho salmon.^8^ The cumulative impact of stormwater runoff on juvenile fish prior to outmigration is difficult to quantify in the field due to their small size and cryptic behaviors. The extent to which survivors from a given storm pulse are vulnerable to a subsequent pulse could be a focus of future research. In addition to the potential for acute mortality, pulsed exposures of developing coho salmon embryos to collected runoff^48^ or 6PPD-quinone^49^ resulted in sublethal impairments to embryo growth and eye development. Sublethal effects noted for susceptible species also include yolk sac edema, blood pooling, reduced swimming performance, irregular hematocrit and/or spinal abnormalities.^39,50^ Therefore, the potential loss of earlier life stages, including outcomes related to juvenile development and/or mortality represents a possible barrier to recovery for these populations. Investigating the integrated effects of real-time runoff exposure throughout early life development would help to understand life cycle impacts and population outcomes for fish developing in roadway-impacted systems.

Clearly, roadway runoff treatment and prevention of particles entering waterways are needed to protect water quality in roadway adjacent habitats, especially those with sensitive species and ecosystems. However, given the widespread nature of roadway runoff, substantial volumes, and expensive costs of treatment, mitigation of 6PPDQ concentrations in habitats of sensitive species is not practical as the only approach. Given the substantial societal resources and efforts spent on salmonid habitat restoration and population supplementation, reformulation of tire rubber chemical compositions to reduce or eliminate chemicals with toxic attributes is both a cost-effective and technically efficient strategy to protect aquatic ecosystems and enhance salmonid health. There remains a need for continued assessment of occurrence, concentration, and mass dynamics of tire rubber and vehicle-derived contaminants in watersheds highly affected by roadway runoff, even those far from “urban centers”, especially as we better define the effect of transient water quality degradation on ecosystem health.

There also exist anecdotal observations of coho salmon juveniles that survive the entire rearing year in highly runoff-impacted watersheds. Considering losses to predation and other natural fates of juveniles in freshwater and subadults in ocean habitats, it may be unreasonable to expect that populations of coho salmon resistant to 6PPDQ would evolve in impacted habitats. The possibility of genetic selection among surviving coho salmon or resistance conferred by sublethal exposure could be explored in future studies.

## Supplementary Material





## Data Availability

Data are available from Washington State University. Contact Jenifer McIntyre (jen.mcintyre@wsu.edu) for further information.

## Notes

Photo of the field facility, the hydrograph for the study period, concentrations for each storm of PPDs, PPD transformation products, other vehicle-derived chemicals, and the eDNA survey results (PDF)
Quality assurance and quality control metrics, concentrations of PPDs, PPDQs, and other vehicle-related chemicals in all water samples, basic water quality in fish exposure waters, the duration that 6PPDQ concentrations were above toxicity thresholds, and peak concentrations of target chemicals for each storm (XLSX)

## References

[1] Walsh, C. J. ; Roy, A. H. ; Feminella, J. W. ; Cottingham, P. D. ; Groffman, P. M. ; Morgan, R. P., II The Urban Stream Syndrome: Current Knowledge and the Search for a Cure J. North Am. Benthological Soc. 2005 10.1899/04-028.1.

[2] LeFevre G. H., Paus K. H., Natarajan P., Gulliver J. S., Novak P. J., Hozalski R. M. (2015). Review of Dissolved Pollutants in Urban Storm Water
and Their Removal and Fate in Bioretention Cells. J. Environ. Eng..

[3] Zgheib S., Moilleron R., Chebbo G. (2012). Priority Pollutants in Urban Stormwater:
Part 1 - Case of Separate Storm Sewers. Water
Res..

[4] Masoner J. R., Kolpin D. W., Cozzarelli I. M., Barber L. B., Burden D. S., Foreman W. T., Forshay K. J., Furlong E. T., Groves J. F., Hladik M. L., Hopton M. E., Jaeschke J. B., Keefe S. H., Krabbenhoft D. P., Lowrance R., Romanok K. M., Rus D. L., Selbig W. R., Williams B. H., Bradley P. M. (2019). Urban Stormwater:
An Overlooked Pathway of Extensive Mixed Contaminants to Surface and
Groundwaters in the United States. Environ.
Sci. Technol..

[5] McIntyre J. K., Lundin J. I., Cameron J. R., Chow M. I., Davis J. W., Incardona J. P., Scholz N. L. (2018). Interspecies Variation in the Susceptibility
of Adult Pacific Salmon to Toxic Urban Stormwater Runoff. Environ. Pollut..

[6] Scholz N. L., Myers M. S., McCarthy S. G., Labenia J. S., McIntyre J. K., Ylitalo G. M., Rhodes L. D., Laetz C. A., Stehr C. M., French B. L., McMillan B., Wilson D., Reed L., Lynch K. D., Damm S., Davis J. W., Collier T. K. (2011). Recurrent
Die-Offs of Adult Coho Salmon Returning to Spawn in Puget Sound Lowland
Urban Streams. PLoS One.

[7] Feist B. E., Buhle E. R., Arnold P., Davis J. W., Scholz N. L. (2011). Landscape
Ecotoxicology of Coho Salmon Spawner Mortality in Urban Streams. PLoS One.

[8] Feist B. E., Buhle E. R., Baldwin D. H., Spromberg J. A., Damm S. E., Davis J. W., Scholz N. L. (2017). Roads to Ruin: Conservation
Threats to a Sentinel Species across an Urban Gradient. Ecol. Appl..

[9] Spromberg J. A., Scholz N. L. (2011). Estimating the Future
Decline of Wild Coho Salmon Populations
Resulting from Early Spawner Die-Offs in Urbanizing Watersheds of
the Pacific Northwest, USA. Integr Environ.
Assess. Manage..

[10] Peter K. T., Tian Z., Wu C., Lin P., White S., Du B., McIntyre J. K., Scholz N. L., Kolodziej E. P. (2018). Using High-Resolution
Mass Spectrometry to Identify Organic Contaminants Linked to Urban
Stormwater Mortality Syndrome in Coho Salmon. Environ. Sci. Technol..

[11] Tian, Z. ; Zhao, H. ; Peter, K. T. ; Gonzalez, M. ; Wetzel, J. ; Wu, C. ; Hu, X. ; Prat, J. ; Mudrock, E. ; Hettinger, R. ; Cortina, A. E. ; Biswas, R. G. ; Vinicius, F. ; Kock, C. ; Soong, R. ; Jenne, A. ; Du, B. ; Hou, F. ; He, H. ; Lundeen, R. ; Gilbreath, A. ; Sutton, R. ; Scholz, N. L. ; Davis, J. W. ; Dodd, M. C. ; Simpson, A. ; Mcintyre, J. K. ; Kolodziej, E. P. A Ubiquitous Tire Rubber-Derived Chemical Induces Acute Mortality in Coho Salmon 2021 https://www.science.org. 10.1126/science.abd6951 33273063

[12] McIntyre J. K., Prat J., Cameron J., Wetzel J., Mudrock E., Peter K. T., Tian Z., Mackenzie C., Lundin J., Stark J. D., King K., Davis J. W., Kolodziej E. P., Scholz N. L. (2021). Treading Water: Tire Wear Particle
Leachate Recreates an Urban Runoff Mortality Syndrome in Coho but
Not Chum Salmon. Environ. Sci. Technol..

[13] Cataldo F. (2018). Early Stages
of P-Phenylenediamine Antiozonants Reaction with Ozone: Radical Cation
and Nitroxyl Radical Formation. Polym. Degrad.
Stab..

[14] Martin, F. ; Sheridan The Vanderbilt Rubber Handbook, 14th ed.; R.T Vanderbilt Company, Inc.: Norwalk, CT, 2010.

[15] Johannessen C., Helm P., Lashuk B., Yargeau V., Metcalfe C. D. (2022). The Tire
Wear Compounds 6PPD-Quinone and 1,3-Diphenylguanidine in an Urban
Watershed. Arch. Environ. Contam. Toxicol..

[16] Cataldo F. (2019). Protection
Mechanism of Rubbers from Ozone Attack. Ozone
Sci. Eng..

[17] Hu X., Zhao H. N., Tian Z., Peter K. T., Dodd M. C., Kolodziej E. P. (2022). Transformation Product Formation upon Heterogeneous
Ozonation of the Tire Rubber Antioxidant 6PPD (N-(1,3dimethylbutyl)-N′-Phenyl-p-Phenylenediamine). Environ. Sci. Technol. Lett..

[18] Hiki K., Yamamoto H. (2022). Concentration and Leachability
of N-(1,3-Dimethylbutyl)-N′-Phenyl-p-Phenylenediamine
(6PPD) and Its Quinone Transformation Product (6PPD-Q) in Road Dust
Collected in Tokyo, Japan. Environ. Pollut..

[19] Hu X., Zhao H., Tian Z., Peter K. T., Dodd M. C., Kolodziej E. P. (2023). Chemical
Characteristics, Leaching, and Stability of
the Ubiquitous Tire Rubber-Derived Toxicant 6PPD-Quinone. Environ. Sci. Process Impacts.

[20] Benis K. Z., Behnami A., Minaei S., Brinkmann M., McPhedran K. N., Soltan J. (2023). Environmental Occurrence
and Toxicity
of 6PPD Quinone, an Emerging Tire Rubber-Derived Chemical: A Review. Environ. Sci. Technol. Lett..

[21] Mayer P. M., Moran K. D., Miller E. L., Brander S. M., Harper S., Garcia-Jaramillo M., Carrasco-Navarro V., Ho K. T., Burgess R. M., Hampton L. M. T., Granek E. F., McCauley M., McIntyre J. K., Kolodziej E. P., Hu X., Williams A. J., Beckingham B. A., Jackson M. E., Sanders-Smith R. D., Fender C. L., King G. A., Bollman M., Kaushal S. S., Cunningham B. E., Hutton S. J., Lang J., Goss H. V., Siddiqui S., Sutton R., Lin D., Mendez M. (2024). Where the
Rubber Meets
the Road: Emerging Environmental Impacts of Tire Wear Particles and
Their Chemical Cocktails. Sci. Total Environ..

[22] Jaeger A., Monaghan J., Tomlin H., Atkinson J., Gill C. G., Krogh E. T. (2024). Intensive Spatiotemporal
Characterization of the Tire
Wear Toxin 6PPD Quinone in Urban Waters. ACS
ES&T Water.

[23] Rauert C., Charlton N., Okoffo E. D., Stanton R. S., Agua A. R., Pirrung M. C., Thomas K. V. (2022). Concentrations of Tire Additive Chemicals
and Tire Road Wear Particles in an Australian Urban Tributary. Environ. Sci. Technol..

[24] Kryuchkov F., Foldvik A., Sandodden R., Uhlig S. (2023). Presence of 6PPD-Quinone
in Runoff Water Samples from Norway Using a New LC–MS/MS Method. Front. Environ. Chem..

[25] Cao G., Wang W., Zhang J., Wu P., Zhao X., Yang Z., Hu D., Cai Z. (2022). New Evidence of Rubber-Derived
Quinones in Water, Air, and Soil. Environ. Sci.
Technol..

[26] Siddiqui, S. ; Andrew James, C. Chemicals of Emerging Concern in Salmon Spawning and Rearing Habitat, 2024. www.kingcounty.gov/Environmentalsceince.

[27] Tian Z., Gonzalez M., Rideout C. A., Zhao H. N., Hu X., Wetzel J., Mudrock E., James C. A., McIntyre J. K., Kolodziej E. P. (2022). 6PPD-Quinone: Revised Toxicity Assessment and Quantification
with a Commercial Standard. Environ. Sci. Technol.
Lett..

[28] Helm P. A., Raby M., Kleywegt S., Sorichetti R. J., Arabian G., Smith D., Howell E. T., Thibeau J. (2024). Assessment
of Tire-Additive Transformation Product 6PPD-Quinone in Urban-Impacted
Watersheds. ACS ES&T Water.

[29] Peter K. T., Hou F., Tian Z., Wu C., Goehring M., Liu F., Kolodziej E. P. (2020). More Than
a First Flush: Urban Creek Storm Hydrographs
Demonstrate Broad Contaminants. Environ. Sci.
Technol..

[30] EPA, U. Acute Aquatic Life Screening Value for 6PPD-Quinone in Freshwater. 2024.

[31] Zhao H. N., Peter K. T., Gonzalez M., Rideout C. A., Hu X., Tian Z., Kolodziej E. P. (2025). Temporal
Dynamics of PPD-Class Antioxidants
and Transformation Products in a Small Roadway-Runoff-Impacted Watershed. Environ. Sci. Technol..

[32] Shankar P., Dalsky E. M., Salzer J. E., Lane R. F., Hammond S., Batts W. N., Gregg J. L., Greer J. B., Kurath G., Hershberger P. K., Hansen J. D. (2025). Evaluation of 6PPD-Quinone Lethal
Toxicity and Sublethal Effects on Disease Resistance and Swimming
Performance in Coastal Cutthroat Trout (*Oncorhynchus clarkii
clarkii*). Environ. Sci. Technol..

[33] Hou F., Tian Z., Peter K. T., Wu C., Gipe A. D., Zhao H., Alegria E. A., Liu F., Kolodziej E. P. (2019). Quantification
of Organic Contaminants in Urban Stormwater by Isotope Dilution and
Liquid Chromatography-Tandem Mass Spectrometry. Anal. Bioanal. Chem..

[34] Chow M. I., Lundin J. I., Mitchell C. J., Davis J. W., Young G., Scholz N. L., McIntyre J. K. (2019). An Urban
Stormwater Runoff Mortality
Syndrome in Juvenile Coho Salmon. Aquat. Toxicol..

[35] Carpenter C. M. G., Wong L. Y. J., Johnson C. A., Helbling D. E. (2019). Fall Creek Monitoring
Station: Highly Resolved Temporal Sampling to Prioritize the Identification
of Nontarget Micropollutants in a Small Stream. Environ. Sci. Technol..

[36] Washington State Legislature, WAC 173–201A-240, 2024.

[37] Lo B. P., Marlatt V. L., Liao X., Reger S., Gallilee C., Ross A. R. S., Brown T. M. (2023). Acute Toxicity
of 6PPD-Quinone to
Early Life Stage Juvenile Chinook (*Oncorhynchus tshawytscha*) and Coho (*Oncorhynchus kisutch*) Salmon. Environ. Toxicol. Chem..

[38] Greer J. B., Dalsky E. M., Lane R. F., Hansen J. D. (2023). Establishing an
in Vitro Model to Assess the Toxicity of 6PPD-Quinone and Other Tire
Wear Transformation Products. Environ. Sci.
Technol. Lett..

[39] Lane R. F., Smalling K. L., Bradley P. M., Greer J. B., Gordon S. E., Hansen J. D., Kolpin D. W., Spanjer A. R., Masoner J. R. (2024). Tire-Derived
Contaminants 6PPD and 6PPD-Q: Analysis, Sample Handling, and Reconnaissance
of United States Stream Exposures. Chemosphere.

[40] Qin H.-p., He K., Fu G. (2016). Modeling Middle
and Final Flush Effects of Urban Runoff
Pollution in an Urbanizing Catchment. J. Hydrol..

[41] Aziz, T. N. ; Mann, A. The Presence and Potential Impacts of the Tire-Wear-Derived Compound (6PPD-q) on NC Aquatic Ecosystems, (FHWA/NC/TA2024–13); North Carolina Department of Transportation Technical Assistance, 2024.

[42] Johannessen C., Helm P., Metcalfe C. D. (2022). Runoff
of the Tire-Wear Compound,
Hexamethoxymethyl-Melamine into Urban Watersheds. Arch. Environ. Contam. Toxicol..

[43] Seitz W., Winzenbacher R. (2017). A Survey on
Trace Organic Chemicals in a German Water
Protection Area and the Proposal of Relevant Indicators for Anthropogenic
Influences. Environ. Monit. Assess..

[44] Challis J. K., Popick H., Prajapati S., Harder P., Giesy J. P., McPhedran K., Brinkmann M. (2021). Occurrences of Tire Rubber-Derived
Contaminants in Cold-Climate Urban Runoff. Environ.
Sci. Technol. Lett..

[45] Liu Y. H., Mei Y. X., Liang X. N., Ge Z. Y., Huang Z., Zhang H. Y., Zhao J. L., Liu A., Shi C., Ying G. G. (2024). Small-Intensity Rainfall Triggers
Greater Contamination
of Rubber-Derived Chemicals in Road Stormwater Runoff from Various
Functional Areas in Megalopolis Cities. Environ.
Sci. Technol..

[46] Losee J. P., Phillips L., Young W. C. (2016). Spawn Timing and Redd Morphology
of Anadromous Coastal Cutthroat Trout (*Oncorhynchus clarkii
clarkii*) in a Tributary of South Puget Sound, Washington. North Am. J. Fish. Manage..

[47] Thomas, P. ; Quinn The Behavior and Ecology of Pacific Salmon and Trout, 1st ed.; De Gruyter Brill, 2005.

[48] McIntyre J. K., Spromberg J., Cameron J., Incardona J. P., Davis J. W., Scholz N. L. (2023). Bioretention Filtration Prevents
Acute Mortality and Reduces Chronic Toxicity for Early Life Stage
Coho Salmon (*Oncorhynchus kisutch*)
Episodically Exposed to Urban Stormwater Runoff. Sci. Total Environ..

[49] Greer J. B., Dalsky E. M., Lane R. F., Hansen J. D. (2023). Tire-Derived Transformation
Product 6PPD-Quinone Induces Mortality and Transcriptionally Disrupts
Vascular Permeability Pathways in Developing Coho Salmon. Environ. Sci. Technol..

[50] Roberts C., Lin J., Kohlman E., Jain N., Amekor M., Alcaraz A. J., Hogan N., Hecker M., Brinkmann M. (2025). Acute and
Subchronic Toxicity of 6PPD-Quinone to Early Life Stage Lake Trout
(*Salvelinus namaycush*). Environ. Sci. Technol..

