# Supplementary material for: Roadway Runoff Induced Acute Mortality in Juvenile Coho Salmon During Spring Storm Events
Source: Environ Sci Technol. 2026 Jan 5;60(2):1723–32. doi: 10.1021/acs.est.5c13992 (PMC12825161; doi:10.1021/acs.est.5c13992)
Supplement: Supplementary file 1 [file es5c13992_si_001.pdf]

Supporting Information

Roadway Runoff Induced Acute Mortality in Juvenile Coho Salmon During Spring Storm Events

Authors: Marlee L. Brown[1,2,3], Nathan Ivy[4], Melissa Gonzalez[1], Justin B. Greer[5], John D. Hansen[5], Edward P. Kolodziej[1,2,3], Jenifer K. McIntyre[4]*

[1]Center for Urban Waters, Tacoma, Washington, 98421 USA

[2]Interdisciplinary Arts and Sciences, University of Washington Tacoma, Tacoma, Washington, 98421 USA

[3]Department of Civil and Environmental Engineering, University of Washington, Seattle, Washington, 98195 USA

[4]Puyallup Research and Extension Center, Washington State University, Puyallup, Washington, 98371 USA

[5]U.S. Geological Survey, Western Fisheries Research Center, Seattle, Washington 98115 USA

Any use of trade, firm, or product names is for descriptive purposes only and does not imply endorsement by the U.S. Government

Manuscript for submission to:

Environmental Science & Technology

15 tables + 16 figures

*Corresponding Author: jen.mcintyre@wsu.edu

Table of Contents: Supporting Information

Supplementary Tables (Excel spreadsheets)
S1: PPDs, PPDQs, and vehicle related chemicals MDLs, MQLs, calibration ranges and ISTD.
S2: QAQC collection dates, type and expected concentrations per storm.
S3: QAQC data for method and field blanks.
S4: PPDs, PPDQs, and vehicle related chemical concentrations in well water.
S5: PPDs, PPDQs, and vehicle related chemical concentrations in creek before and after grabs.
S6: PPDs, PPDQs, and vehicle related chemical spike and recoveries.
S7: Summary of basic daily water quality across the three fish exposures.
S8: PPD concentrations in baseflow sampling.
S9: PPDQ concentrations in baseflow sampling.
S10: Vehicle related chemical concentrations in baseflow sampling.
S11: Hours 6PPDQ concentrations were at or above LC_{50} values.
S12: PPD concentrations in targeted storms.
S13: PPDQ concentrations in targeted storms.
S14: Vehicle related chemical concentrations in targeted storms.
S15: Per-storm peak concentrations and mass loads for all PPDs, PPDQs, and vehicle related chemicals.

Supplementary Figures (this PDF file)
S1: Miller Creek Field Laboratory Facility
S2: Miller Creek hydrograph for April-June 2024.
S3: PPD concentrations and discharge for Storm 1.
S4: PPD concentrations and discharge for Storm 2.
S5: PPD concentrations and discharge for Storm 3.
S6: Pollutographs for PPD antioxidants across the three storms.
S7: PPD transformation products concentrations and discharge for Storm 1.
S8: PPD transformation products concentrations and discharge for Storm 2.
S9: PPD transformation products concentrations and discharge for Storm 3.
S10: Pollutographs for PPD transformation products across the three storms.
S11: Vehicle-derived chemicals concentrations and discharge for Storm 1.
S12: Vehicle-derived chemicals concentrations and discharge for Storm 2.
S13: Vehicle-derived chemicals and discharge for Storm 3.
S14: Pollutographs for vehicle-derived chemicals across the three storms.
S15: Concentrations of 1,3 DMBA and discharge for Storms 1-3.
S16: Results of eDNA survey of Miller Creek.

Number of pages: 11

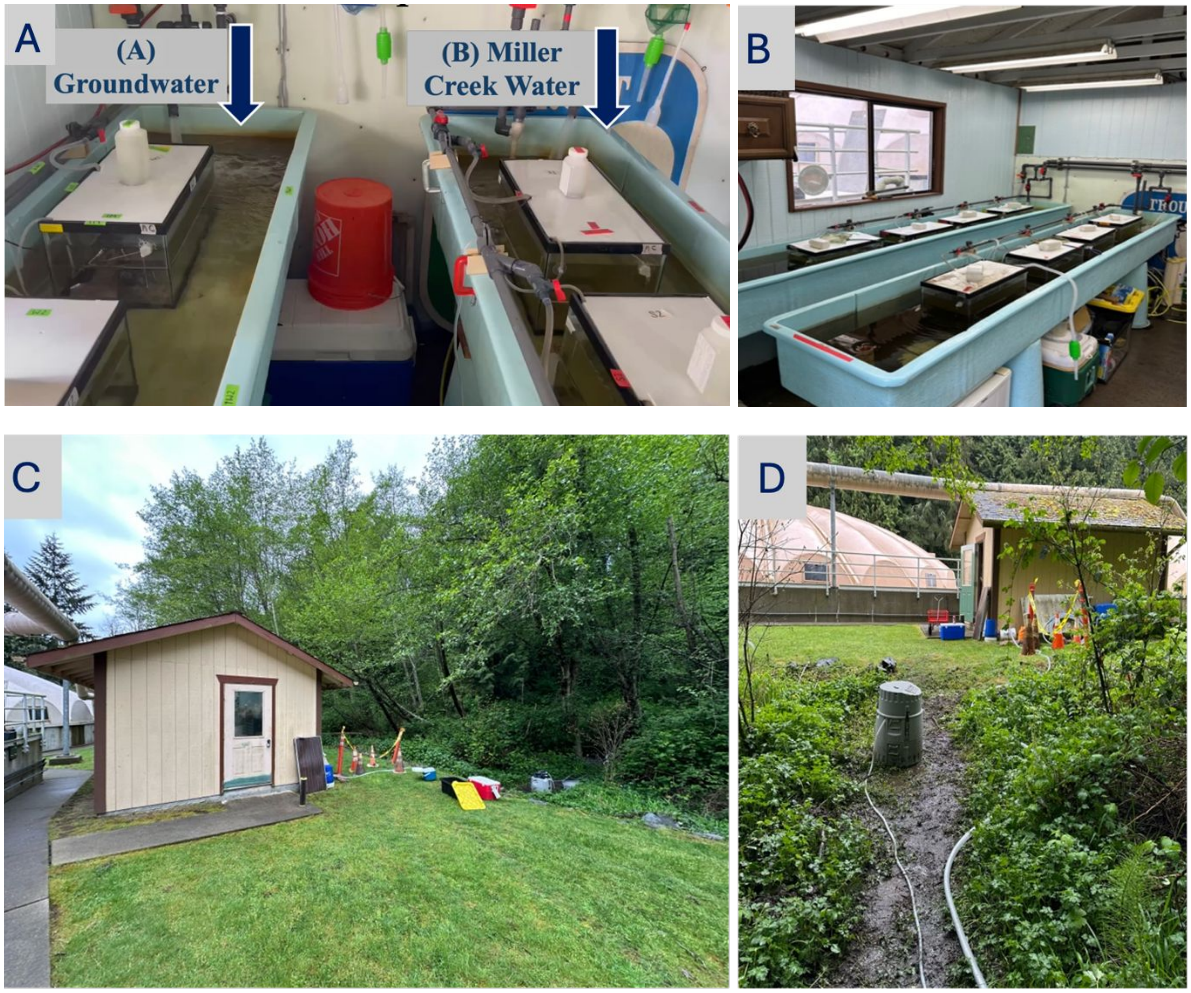

Figure S1. Field laboratory facility on Miller Creek used for real time exposures of juvenile coho to targeted storms. (A) Groundwater control and Miller Creek exposures in side-by-side raceways; (B) exposure system of four fish tanks per raceway per storm; (C) field laboratory facility; and (D) automated ISCO sampler and exposure water feed. Photos of sampling site and facility were from different days.

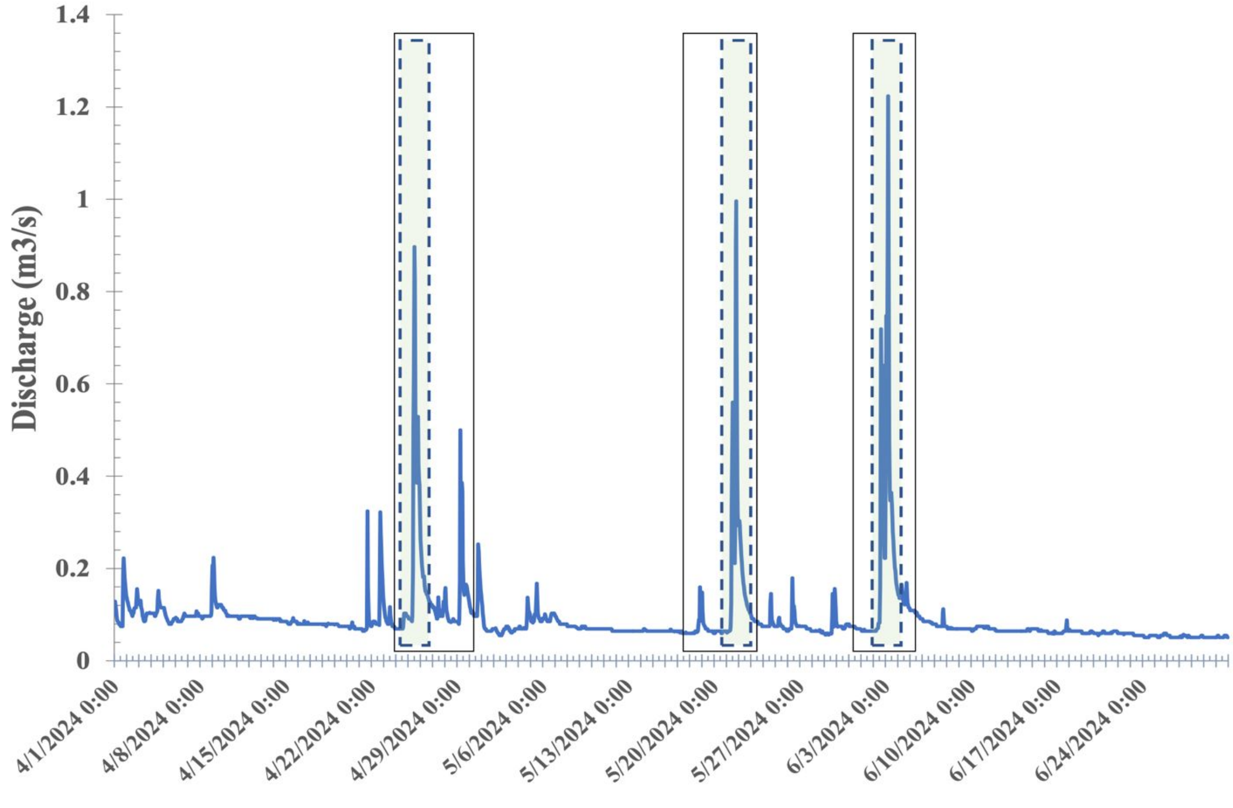

Figure S2. Hydrograph (as discharge in m^3/s, measured at King County gage 42a) of Miller Creek (Burien/Normandy Park, WA, USA) over the period April 1 - June 30, 2024. Shaded boxes with dashed lines indicate the three storm events targeted by automatic sampling and the solid rectangles when juvenile coho salmon exposure studies were conducted.

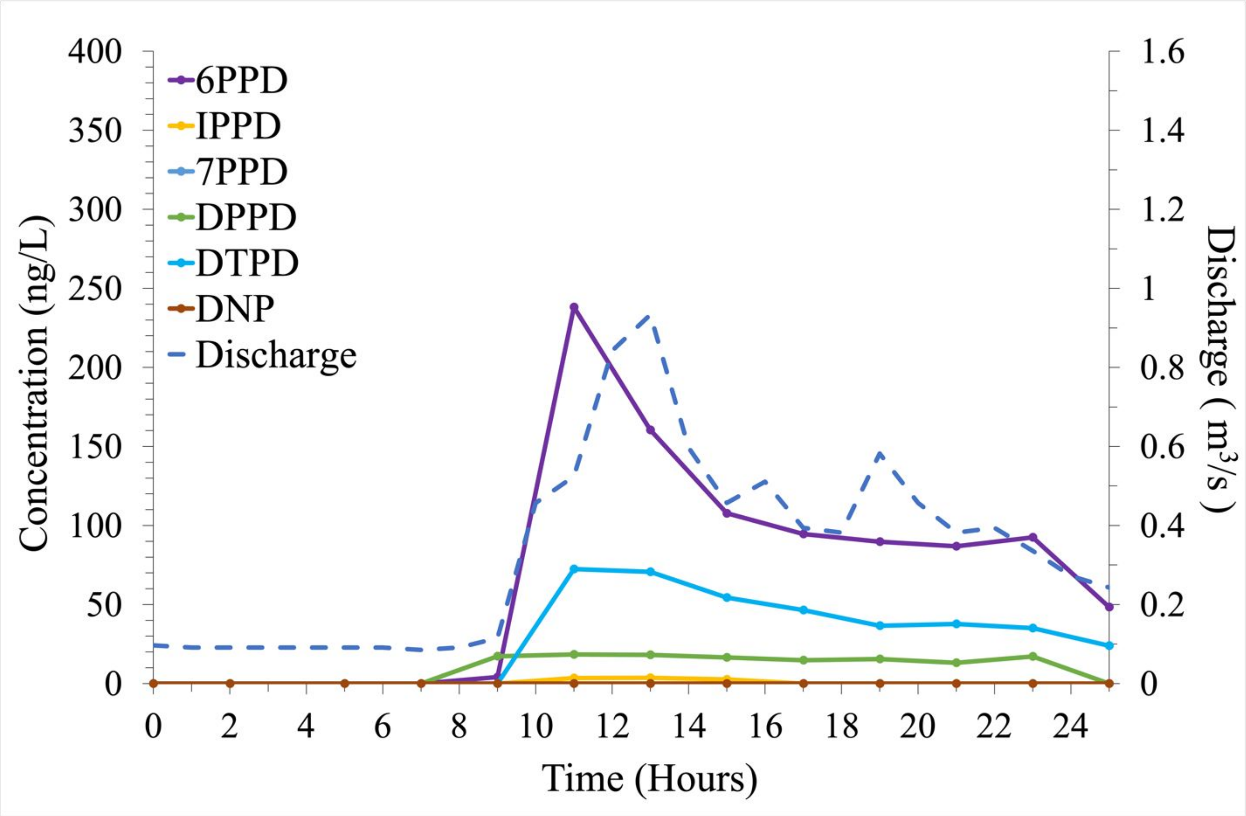

Figure S3. Concentrations of PPD antioxidants (ng/L) and stream discharge (m^3/s) in Miller Creek (Burien/Normandy Park, WA, USA) during Storm 1 (April 25, 2024). Discharge was measured at King County gage 42a (47.44548, -122.35196; King County Hydrologic Monitoring Program).

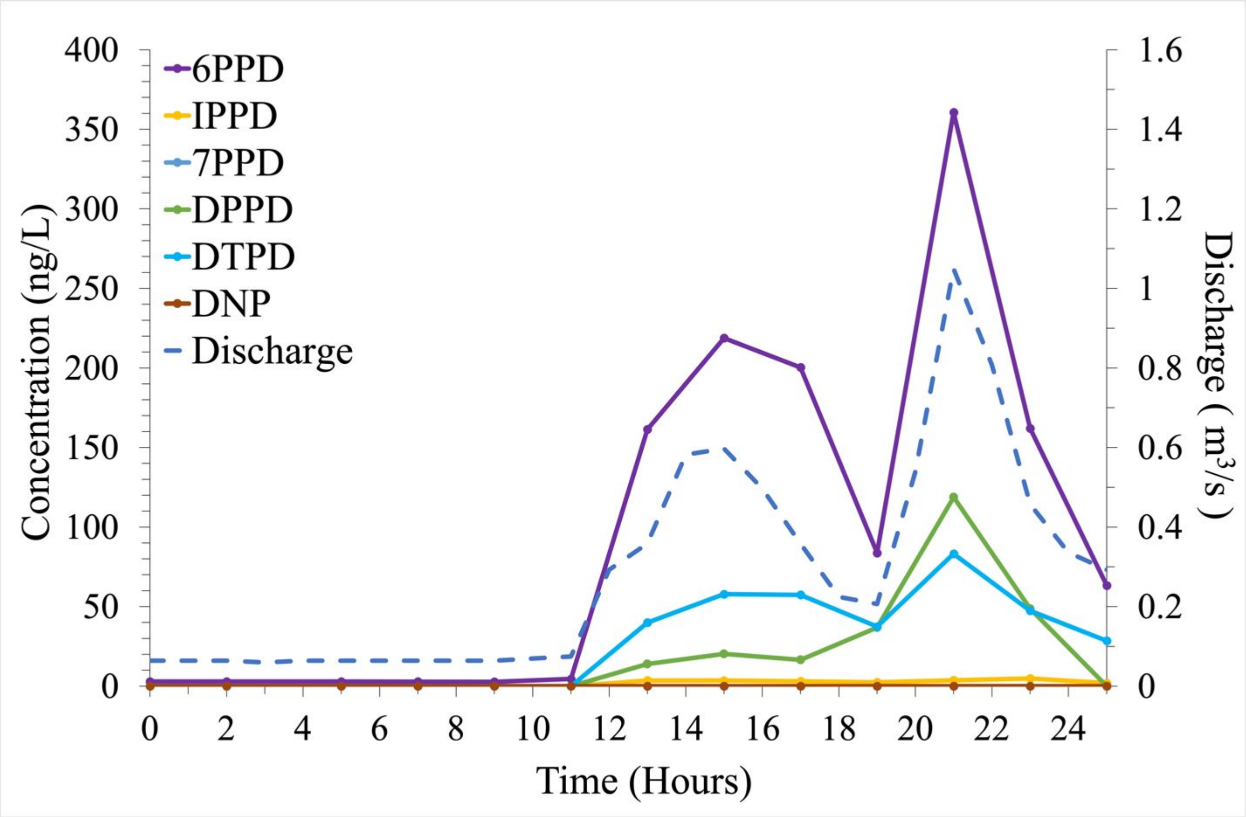

Figure S4. Concentrations of PPD antioxidants (ng/L) and stream discharge (m^3/s) in Miller Creek (Burien/Normandy Park, WA, USA) during Storm 2 (May 21, 2024). Discharge was measured at King County gage 42a (47.44548, -122.35196; King County Hydrologic Monitoring Program).

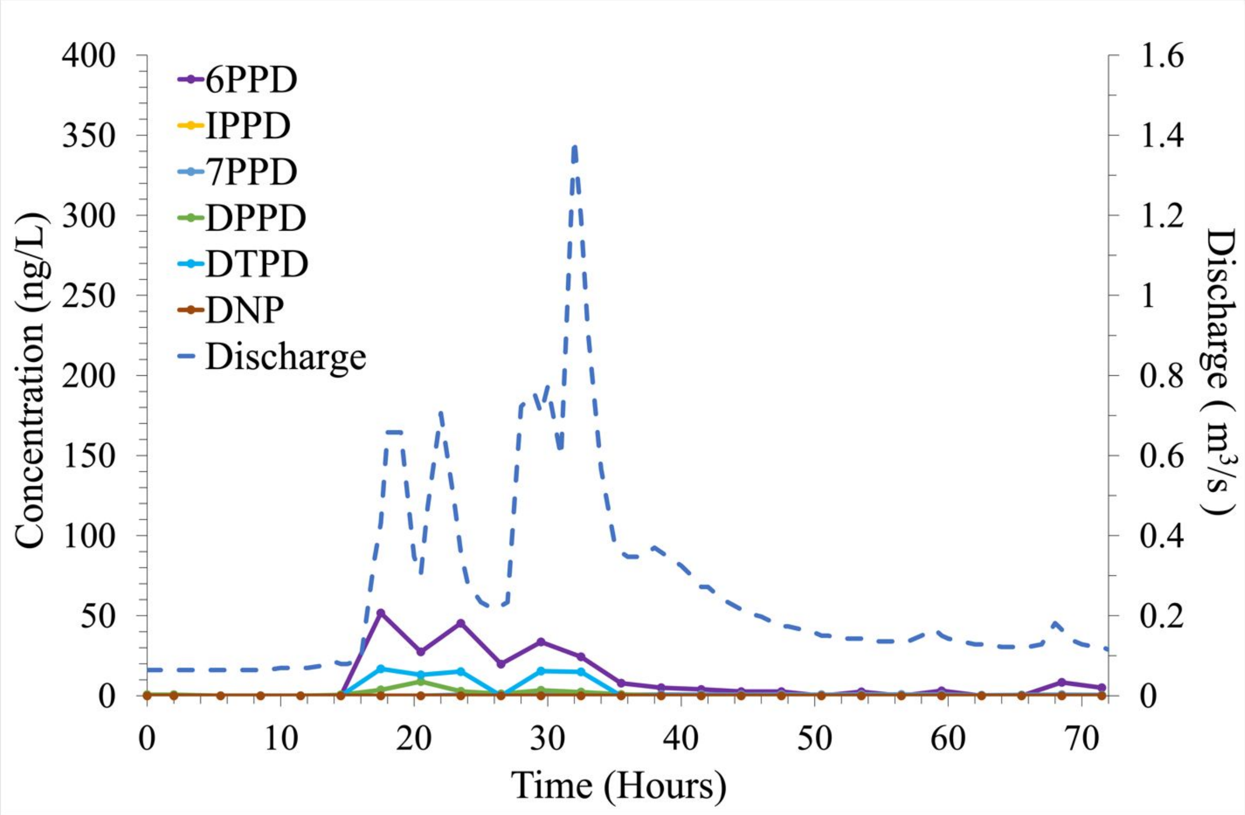

Figure S5. Concentrations of PPD antioxidants (ng/L) and stream discharge (m^3/s) in Miller Creek (Burien/Normandy Park, WA, USA) during Storm 3 (June 1-4, 2024). Discharge was measured at King County gage 42a (47.44548, -122.35196; King County Hydrologic Monitoring Program).

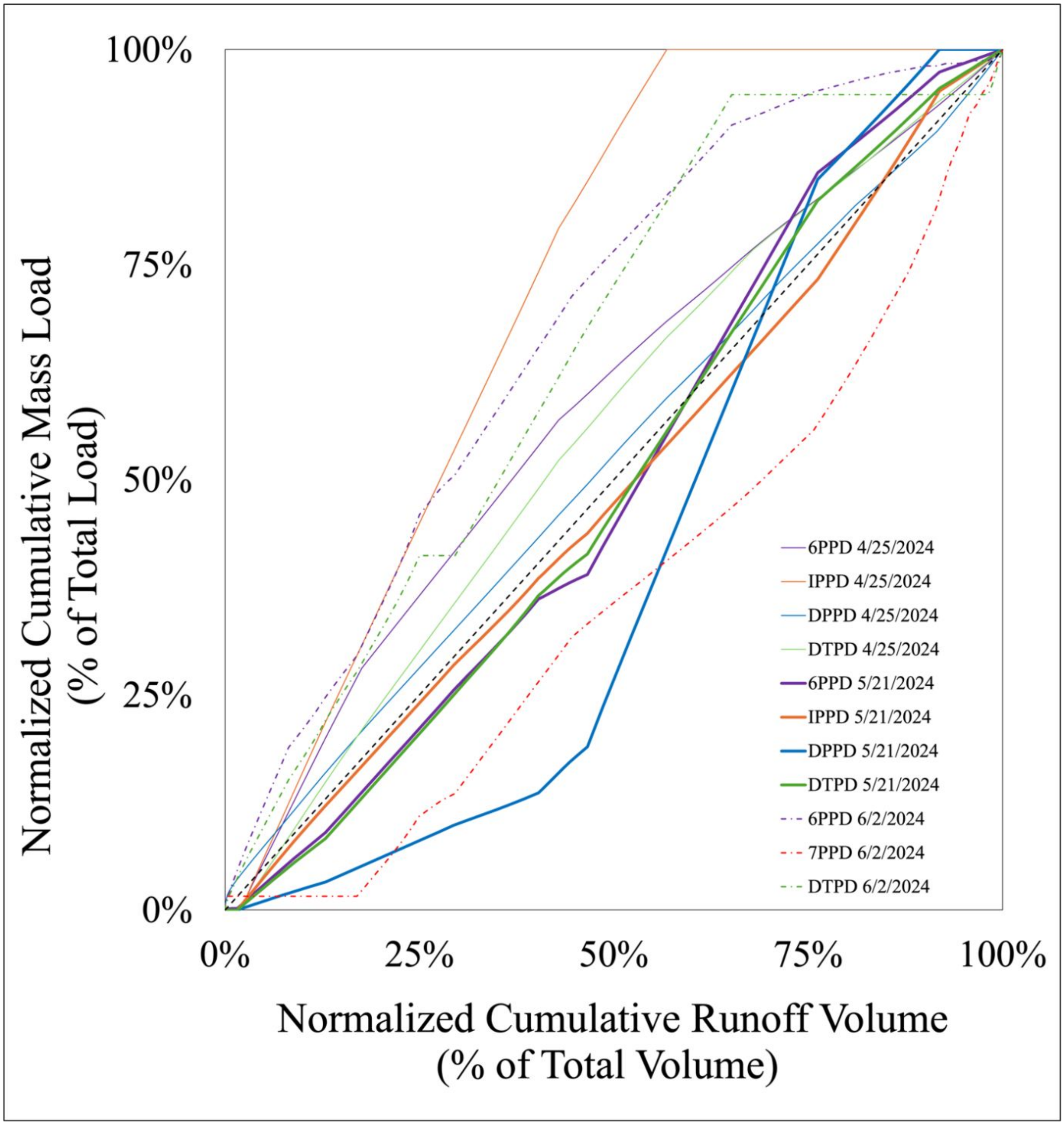

Figure S6. Pollutographs of PPD antioxidants as normalized cumulative mass load (as a % of total mass load), plotted against normalized cumulative runoff volume (as a % of total runoff volume) across the three storm events. The 1:1 mass load to runoff volume curve (dashed line) represents a proportional rate of contaminant mass transport and runoff volume into the receiving water across the storm event.

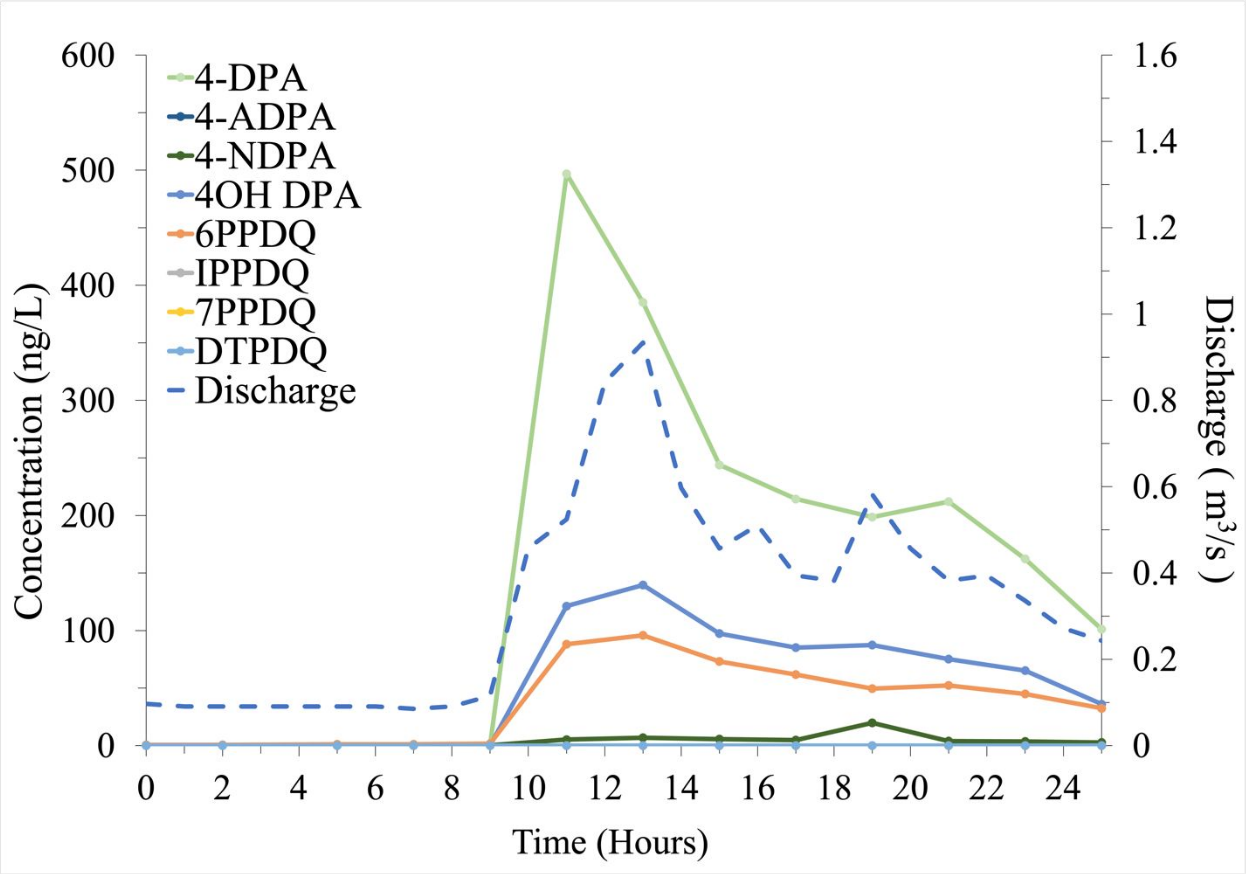

Figure S7. Concentrations of PPD transformation products (ng/L) and stream discharge (m^3/s) in Miller Creek (Burien/Normandy Park, WA, USA) during Storm 1 (April 25, 2024). Discharge was measured at King County gage 42a (47.44548, -122.35196; King County Hydrologic Monitoring Program).

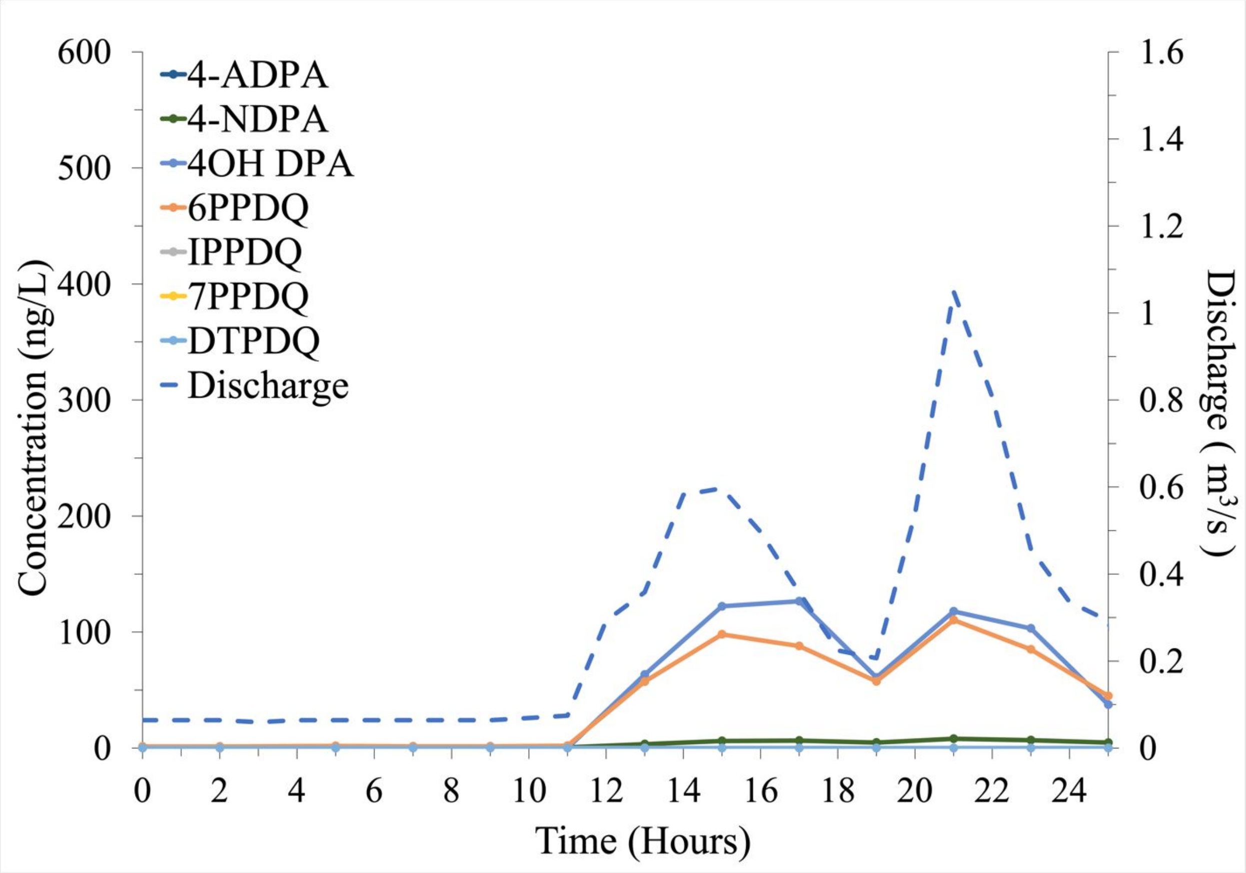

Figure S8. Concentrations of PPD transformation products (ng/L) and stream discharge (m^3/s) in Miller Creek (Burien/Normandy Park, WA, USA) during Storm 2 (May 21, 2024). Discharge was measured at King County gage 42a (47.44548, -122.35196; King County Hydrologic Monitoring Program).

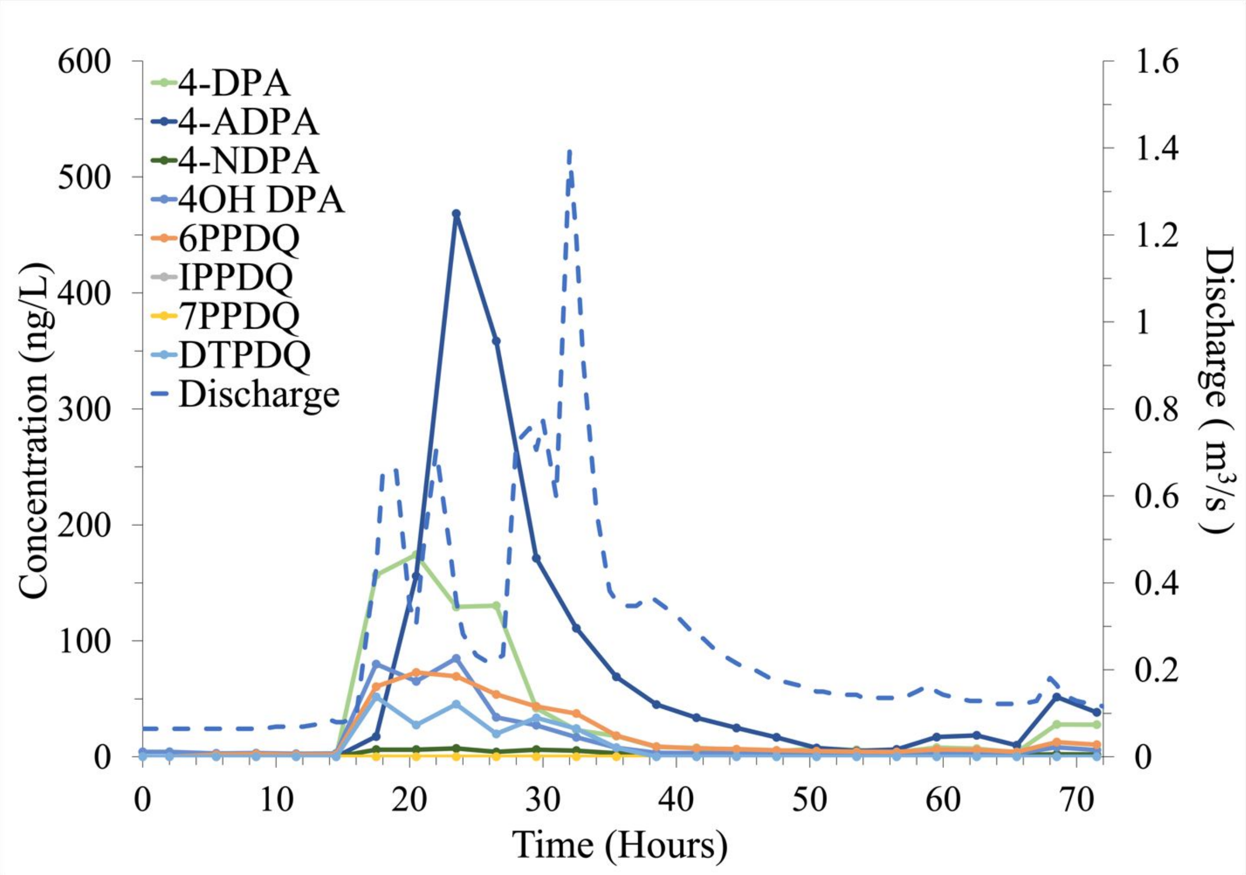

Figure S9. Concentrations of PPD transformation products (ng/L) and stream discharge (m^3/s) in Miller Creek (Burien/Normandy Park, WA, USA) during Storm 3 (June 2, 2024). Discharge was measured at King County gage 42a (47.44548, -122.35196; King County Hydrologic Monitoring Program).

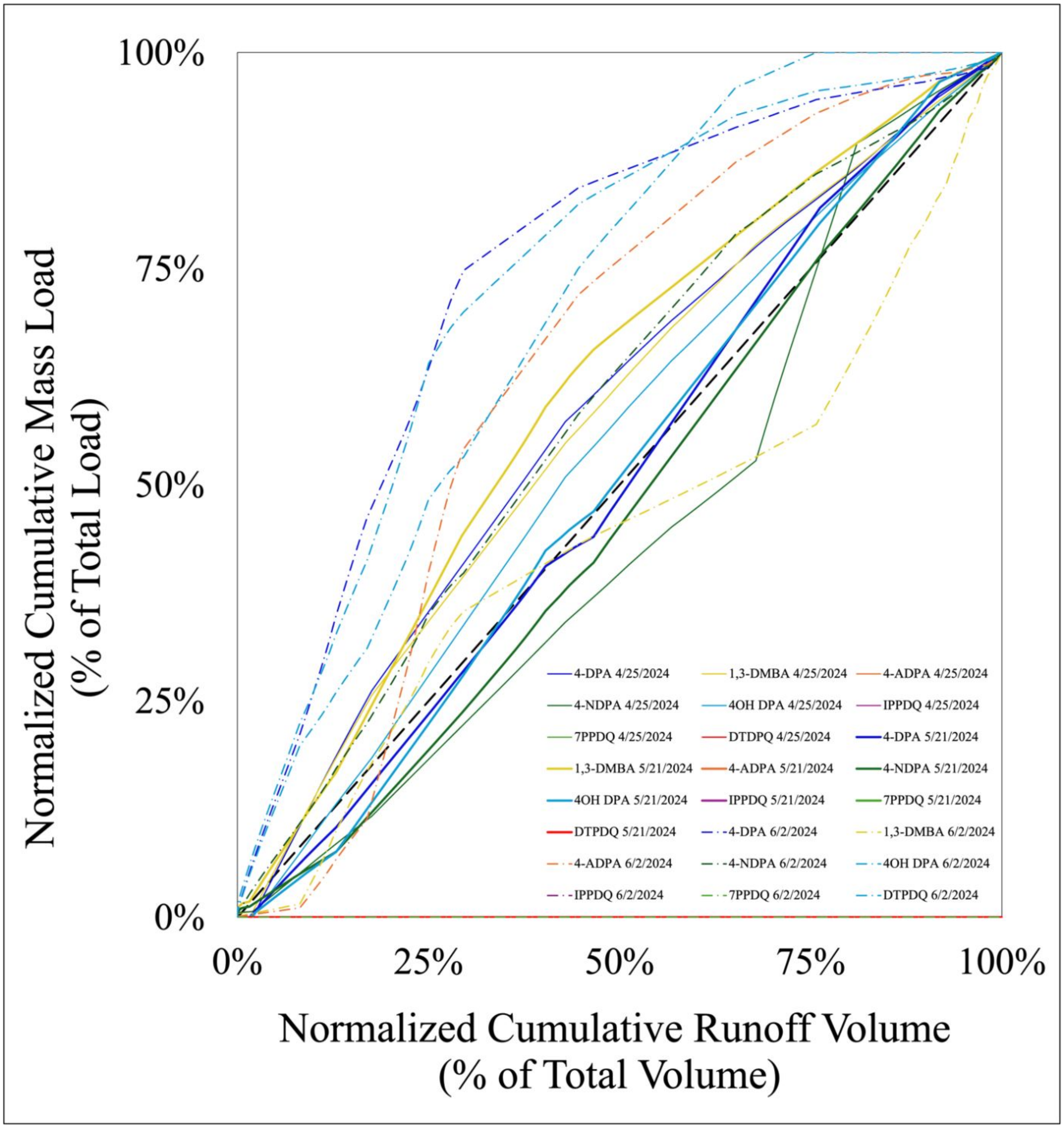

Figure S10. Pollutographs of PPD transformation products as normalized cumulative mass load (as a % of total mass load), plotted against normalized cumulative runoff volume (as a % of total runoff volume) across the three storm events in Miller Creek. The 1:1 mass load to runoff volume curve (dashed line) represents a proportional rate of contaminant mass transport and runoff volume into the receiving water across the storm event.

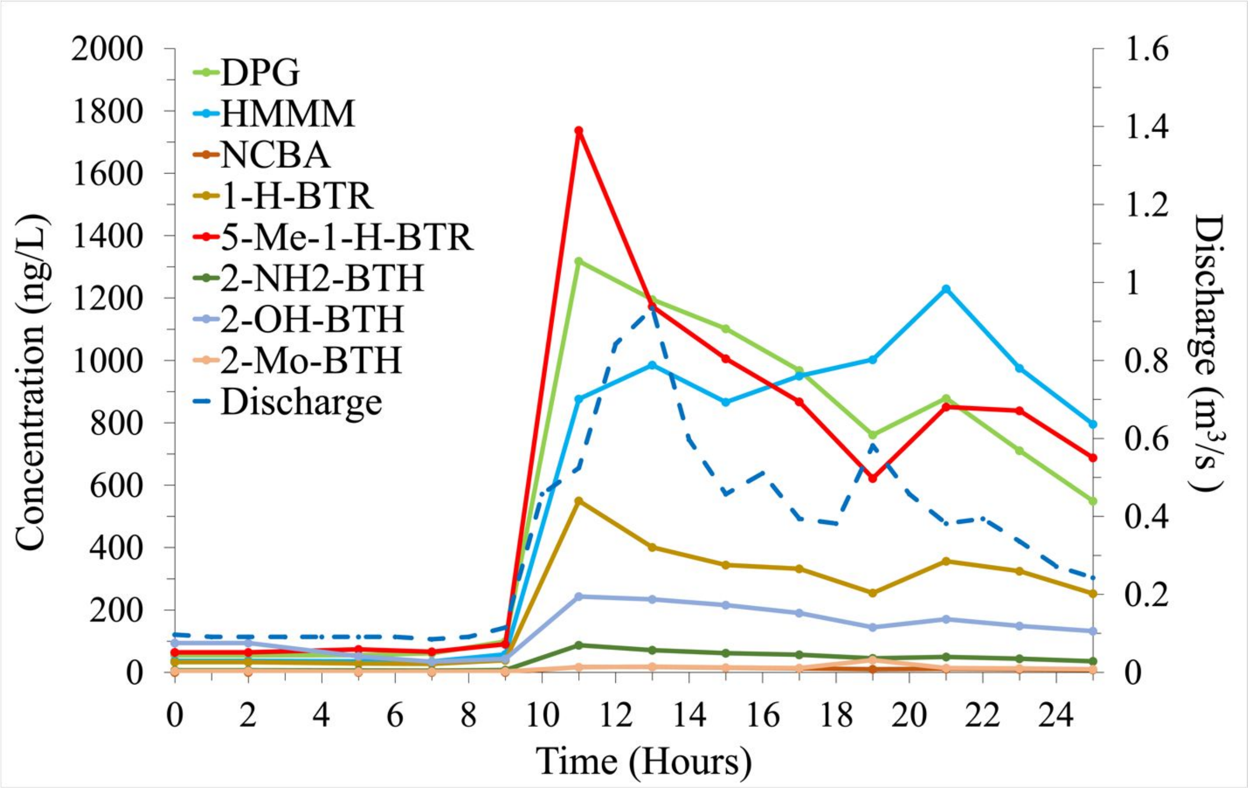

Figure S11. Concentrations of vehicle-derived chemicals (ng/L) and stream discharge (m^3/s) in Miller Creek (Burien/Normandy Park, WA, USA) during Storm 1 (April 25, 2024). Discharge was measured at King County gage 42a (47.44548, -122.35196; King County Hydrologic Monitoring Program).

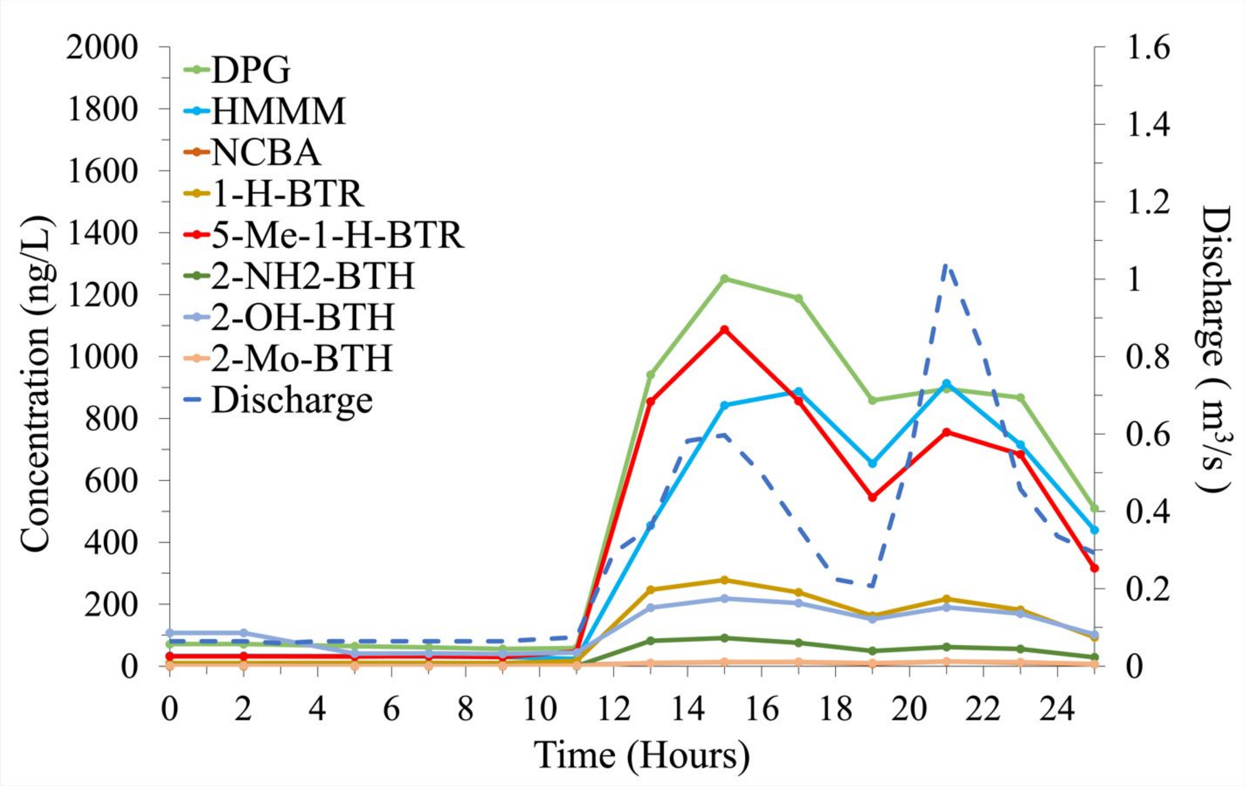

Figure S12. Concentrations of vehicle-derived chemicals (ng/L) and stream discharge (m^3/s) in Miller Creek (Burien/Normandy Park, WA, USA) during Storm 2 (May 21, 2024). Discharge was measured at King County gage 42a (47.44548, -122.35196; King County Hydrologic Monitoring Program).

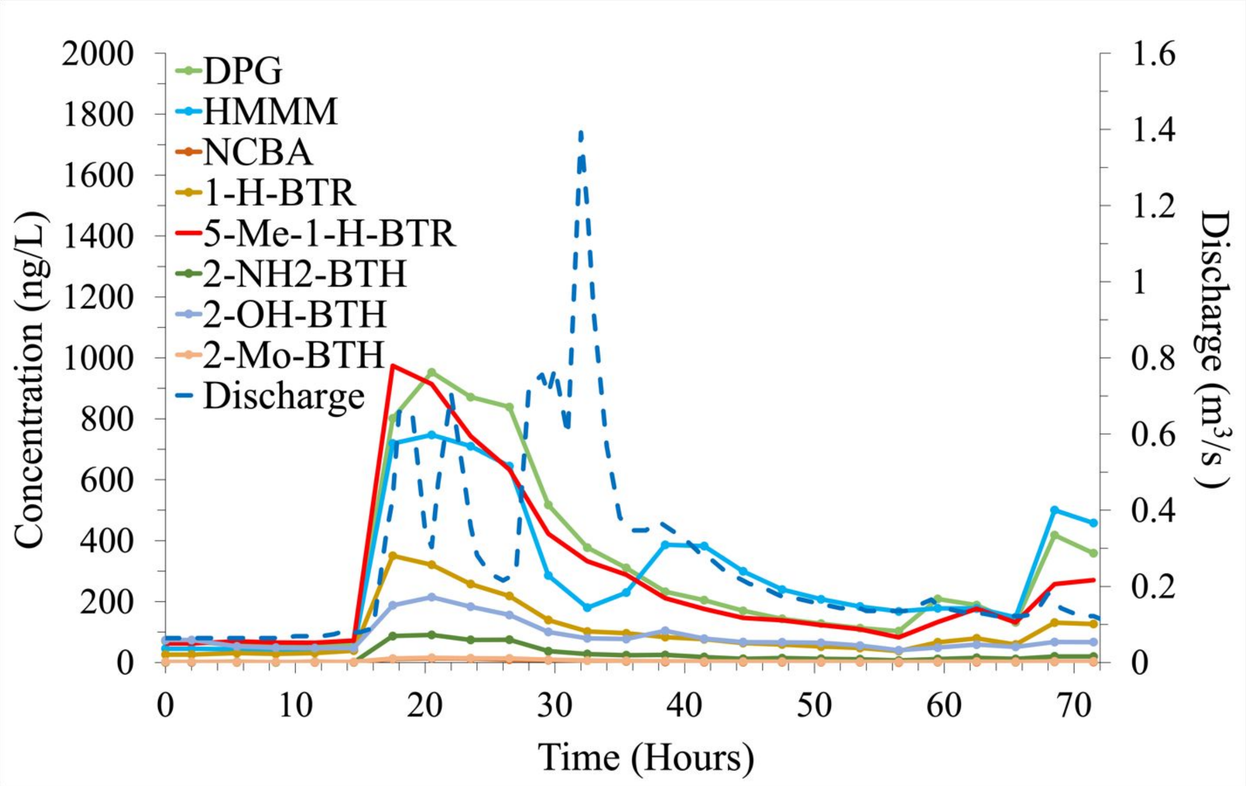

Figure S13. Concentrations of vehicle-derived chemicals (ng/L) and stream discharge (m^3/s) in Miller Creek (Burien/Normandy Park, WA, USA) during Storm 3 (June 2, 2024). Discharge was measured at King County gage 42a (47.44548, -122.35196; King County Hydrologic Monitoring Program).

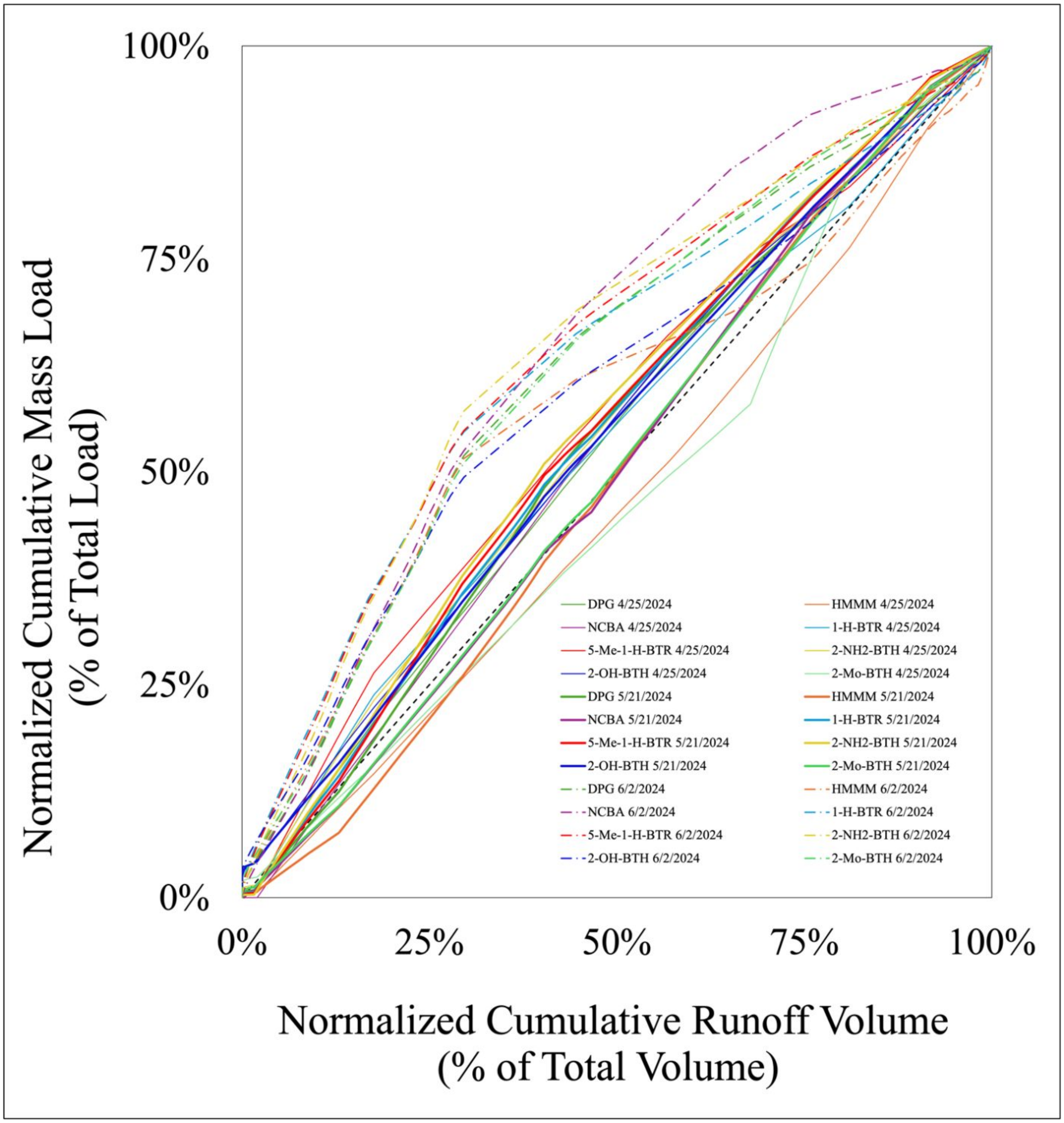

Figure S14. Pollutographs of vehicle-derived chemicals as normalized cumulative mass load (as a % of total mass load), plotted against normalized cumulative runoff volume (as a % of total runoff volume) across the three storm events in Miller Creek. The 1:1 mass load to runoff volume curve (dashed line) represents a proportional rate of contaminant mass transport and runoff volume into the receiving water across the storm event.

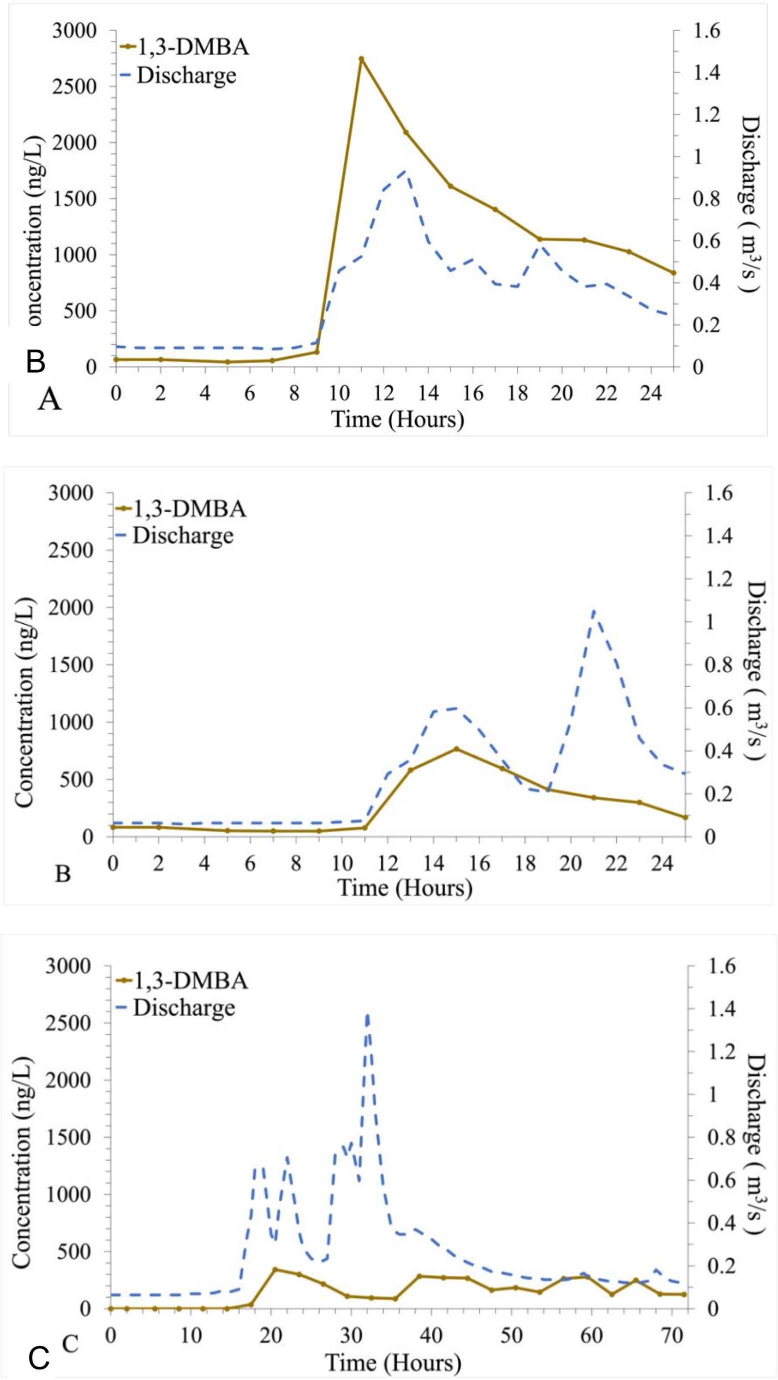

Figure S15. 1,3-DMBA concentrations plotted against discharge volume. (A) Storm 1, April 25, 2024 sampling event, (B) Storm 2, May 21, 2024 sampling event, and (C) Storm 3, June 1 through June 4, 2024 sampling event, 1,3-DMBA concentrations (ng/L) were quantified and plotted on the left y axis, discharge (m^3/s) plotted on the right y axis, both plotted as a time series (note the differing X-axis scales).

A.

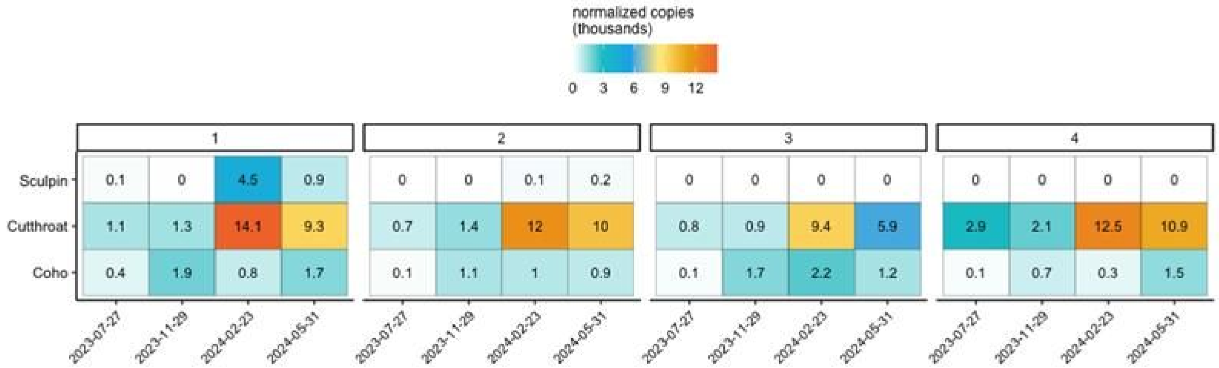

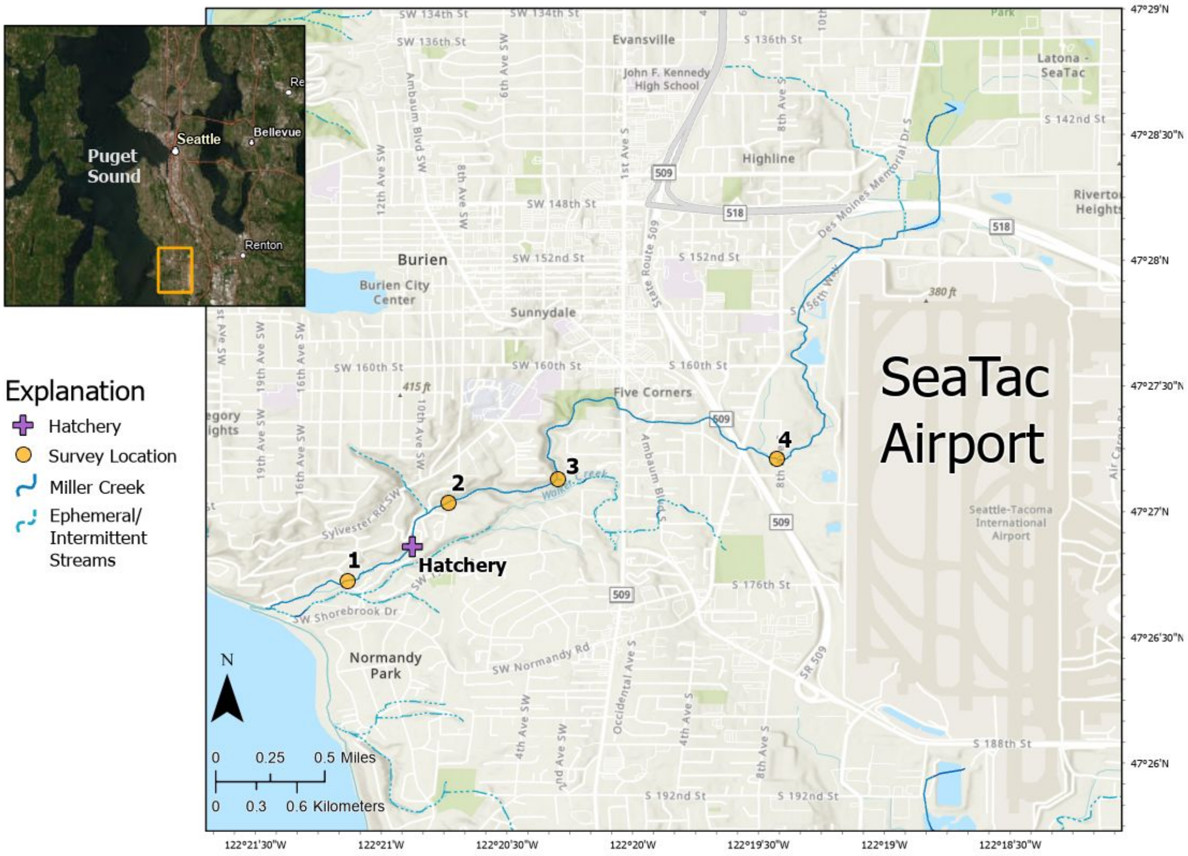

Figure S16. Occupancy and distribution of sculpin, coastal cutthroat trout, chum salmon and coho salmon in Miller Creek, Burien WA via seasonal eDNA analysis. A) Sampling was conducted at the following 4 locations (from left to right): site 1: 47.445463, -122.352172, site 2: 47.450627, -122.345492, site 3: 47.452183, -122.338268, site 4: 47.453543, -122.323759. EDNA survey results are depicted as mean Ct values that were normalized to flow rates for dates sampled. Chum salmon (not shown) were only detected November 2023 and February 2023 at site 1 at low levels consistent with transient lifestyle of chum salmon in freshwater streams and creeks. B) Map depicting eDNA sampling sites and field laboratory location in Burien, WA.

Materials and Methods:

Surveys of eDNA were performed quarterly (July 27, 2023; November 29, 2023; February 23, 2024; and May 31, 2024) at 4 longitudinally spaced sites on Miller Creek. eDNA sampling consisted of collecting 5 L subsurface water samples in triplicate at each site using a SmithRoot eDNA sampler in conjunction with 5 uM PES eDNA self-preserving filters (Smith-Root). Negative field controls consisted of filtering 2 L of laboratory deionized water. Filter samples were processed according to Ostberg et al. (2024). Briefly, filters were removed, cut into 2 mm strips and placed in lysis buffer. DNA was then extracted using Qiagen DNAeasy Blood and Tissue kits (Santa Clarita, California). The PCR workflow consisted of multiple workstations, each dedicated to DNA extractions, preparation of qPCR reagents, preparation of DNA standards, and PCR amplification. All DNA extracts were tested for the presence of PCR inhibition. (1) Inhibited samples were defined as having >1 cycle threshold (Ct) shifts relative to the mean nontemplate control; no samples appeared inhibited.

Four target species were tested: coho salmon (*O. kisutch*), coastal cutthroat trout (*O. clarkii clarkii*), chum salmon (*O. keta*), and the sculpin superfamily (*Cottoidea*). (2,3) PCR assays contained 1× Gene Expression Mastermix (ThermoFisher Scientific, Waltham, Maine), 1× custom TaqMan primer and probe mix (a final concentration of 450 nM for each forward and reverse primers and 125 nM probe) and were run on a ViiA7 Real-Time PCR system (Waltham, MA) using the following default cycle parameters: initial steps of 2 min at 50°C, then 10 min at 95°C, then 45 cycles of denaturing at 95°C for 15 s, and annealing/extension at 60°C for 1 min. Results were analyzed using ViiA 7 RUO 1.2.4 software. Three technical replicates were used per DNA sample; negative controls consisted of field controls, DNA extraction controls, and nontemplate controls. All negative controls yielded no amplification. The limit of detection, quantification and efficiency of each assay was accomplished by utilizing gBlock double-stranded gene fragments (Integrated DNA Technologies, Coralville, Iowa) representing the species amplicon for each assay and performing real-time PCR on a dilution series consisting of 10,000, 1,000, 100, 10, 5, and 1 copies per reaction with 40 replicate samples at each concentration. Mean Ct were normalized using creek data from the sample day and time (KingCounty gauge 42A).

Cited References

1. Duda, J. J., Hoy, M. S., Chase, D. M., Pess, G. R., Brenkman, S. J., McHenry, M. M., & Ostberg, C. O. Environmental DNA is an effective tool to track recolonizing migratory fish following large-scale dam removal. *Environmental DNA*. 2021, 3(1), 121-141, DOI:10.1002/edn3.134
2. Hoy, M. S., & Ostberg, C. O. (2020). Development of two quantitative PCR assays for detection of several Cottus species from environmental DNA in Pacific coast watersheds of North America. *Conservation Genetics Resources*, 12(3), 361-363. DOI:10.1007/s12686-019-01118-7
3. Ostberg, C.O., Pier, C., Chase, D.M., & Perry, R.W. Spatial and temporal surveys of salmon environmental (eDNA) in a Seattle Urban Creek. *Northwest Science*. 2024, 97(3) 167-184. DOI:10.3955/046.097.0302.
